# Emerging functions and therapeutic targets of IL‐38 in central nervous system diseases

**DOI:** 10.1111/cns.14550

**Published:** 2024-02-09

**Authors:** Yuan Gao, Luwei Cai, Yulu Wu, Min Jiang, Yidan Zhang, Wenjing Ren, Yirui Song, Lili Li, Ziguang Lei, Youzhuang Wu, Luwen Zhu, Jing Li, Dongya Li, Guohong Li, Chengliang Luo, Luyang Tao

**Affiliations:** ^1^ Department of Forensic Medicine, School of Basic Medicine and Biological Sciences Soochow University Suzhou China; ^2^ Department of Neurosurgery Pennsylvania State University College of Medicine State College Pennsylvania USA; ^3^ Department of Forensic Science Wenzhou Medical University Wenzhou Zhejiang China; ^4^ Department of Forensic Medicine, Tongji Medical College Huazhong University of Science and Technology Wuhan China; ^5^ Department of Child and Adolescent Healthcare Children's Hospital of Soochow University Suzhou China; ^6^ Department of Orthopedics The Affiliated Hospital of Xuzhou Medical University Xuzhou China

**Keywords:** Alzheimer's disease, autism spectrum disorder, IL‐36R, IL‐38, ischemic stroke, neuromyelitis optica disorder, spinal cord injury, traumatic brain injury

## Abstract

Interleukin (IL)‐38 is a newly discovered cytokine of the IL‐1 family, which binds various receptors (i.e., IL‐36R, IL‐1 receptor accessory protein‐like 1, and IL‐1R1) in the central nervous system (CNS). The hallmark physiological function of IL‐38 is competitive binding to IL‐36R, as does the IL‐36R antagonist. Emerging research has shown that IL‐38 is abnormally expressed in the serum and brain tissue of patients with ischemic stroke (IS) and autism spectrum disorder (ASD), suggesting that IL‐38 may play an important role in neurological diseases. Important advances include that IL‐38 alleviates neuromyelitis optica disorder (NMOD) by inhibiting Th17 expression, improves IS by protecting against atherosclerosis via regulating immune cells and inflammation, and reduces IL‐1β and CXCL8 release through inhibiting human microglial activity post‐ASD. In contrast, IL‐38 mRNA is markedly increased and is mainly expressed in phagocytes in spinal cord injury (SCI). IL‐38 ablation attenuated SCI by reducing immune cell infiltration. However, the effect and underlying mechanism of IL‐38 in CNS diseases remain inadequately characterized. In this review, we summarize the biological characteristics, pathophysiological role, and potential mechanisms of IL‐38 in CNS diseases (e.g., NMOD, Alzheimer's disease, ASD, IS, TBI, and SCI), aiming to explore the therapeutic potential of IL‐38 in the prevention and treatment of CNS diseases.

## Graphical abstract

IL‐38 regulates immune and inflammatory responses after NMOD, AD, ASD, IS, TBI, and SCI by competitively binding to IL36R with IL‐36. Briefly, IL‐38 recruits myeloid differentiation primary response 88 (MyD88) by binding to IL‐1R1 or IL‐36R, activating downstream signaling pathways including NF‐κB and MAPK pathways, thereby enhancing the production of pro‐inflammatory cytokines and chemokines. In addition, IL‐38 selectively recruits MEK or upregulates channel protein‐dependent SIRT1 by binding to IL1RAPL1, thereby reducing NF‐κB, AP‐1, and MAPK.

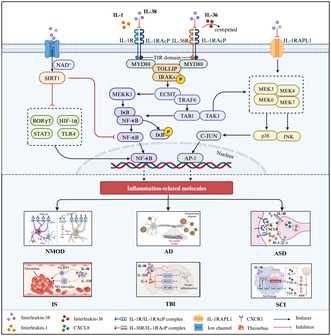

## 1 INTRODUCTION

Central nervous system (CNS) diseases have relatively high morbidity, disability, and mortality rates. Nearly one in six people worldwide suffer from CNS diseases, which imposes a significant economic burden on families and society.^1^, ^2^, ^3^ The hallmark pathological change of CNS diseases is neuroinflammation. A moderate inflammatory response may serve as a neurological defense, whereas excessive or persistent inflammation can be destructive.^4^, ^5^, ^6^ Previous studies have found that the expression levels of interleukin (IL)‐1β, IL‐17, and IL‐36 are significantly elevated in the serum of patients with Alzheimer's disease (AD), ischemic stroke (IS), and traumatic brain injury (TBI), accompanied by severe neurological dysfunction. After effective anti‐inflammatory treatment, the expression levels of these cytokines were remarkably reduced, and cognitive dysfunction was effectively improved.^7^, ^8^, ^9^, ^10^, ^11^, ^12^, ^13^ Of further note, IL‐36 receptor antagonist (IL‐36Rα) and IL‐38, two anti‐inflammatory cytokines with similar functions in IL‐1F reduced neuronal death and improved cognitive function defects by down‐regulating the expression levels of IL‐1β, tumor necrosis factor α (TNF‐α), and IL‐6.^14^, ^15^ Therefore, targeting inflammatory cytokines is a promising new therapy for the treatment of CNS diseases and injuries.

IL‐38 is a newly discovered cytokine in the IL‐1 family and plays an important role in a variety of diseases, including but not limited to CNS diseases.^16^, ^17^, ^18^, ^19^, ^20^, ^21^, ^22^, ^23^, ^24^ IL‐38 is mainly expressed in the brain, heart, lungs, spleen, thymus, tonsils, and skin, and is less distributed in immune‐inactive tissues.^25^, ^26^ Moreover, its protein is mainly synthesized and secreted by fibroblast‐like synoviocytes, keratinocytes, peripheral blood mononuclear cells, and CD19+ B cells.^27^, ^28^ IL‐38 signaling requires the binding of its three receptors, IL‐36R, IL‐1R, and IL‐1 receptor accessory protein‐like 1 (IL‐1RAPL1).^29^, ^30^ When cells undergo apoptosis or necrosis, IL‐38 is secreted through autocrine, paracrine, or endocrine pathways and binds to the receptor IL‐36R and co‐receptor IL‐1R on the surface of its own cells or neighboring cells, thereby exerting corresponding biological activities.^31^ IL‐38 has been shown to have both anti‐inflammatory and pro‐inflammatory properties in CNS diseases. During the acute phase of autism spectrum disorder (ASD) patients, IL‐38 protein expression levels were dramatically elevated in serum and significantly decreased in the hippocampus and amygdala. Of note, IL‐38 inhibits the expression of pro‐inflammatory factors IL‐1β and CXCL8 by reducing the proliferation and migration of microglia, indicating that IL‐38 plays an anti‐inflammatory protective role following ASD and can be utilized as a biological marker for the diagnosis and treatment of ASD patients.^32^ In contrast, the expression level of IL‐38 mRNA is markedly increased and is mainly expressed in F4/80‐positive macrophages and Iba‐1‐positive microglia residing and infiltrating the spinal cord. Moreover, IL‐38 deletion attenuated spinal cord injury (SCI) by reducing inflammation and immune cell infiltration.^17^ Furthermore, numerous studies have found that low concentrations of mature IL‐38 can limit the production of Th17 cytokines by Th17 cells, increase the polarization of macrophages toward M2 macrophages, and inhibit the polarization toward M1 macrophages, thereby reducing the inflammatory response.^19^ Conversely, high concentrations of mature IL‐38 exacerbate the inflammatory response by increasing the expression of pro‐inflammatory factors IL‐6, IL‐1β, and TNF‐α.^33^ Collectively, this evidence suggests that the conflicting functions of IL‐38 in CNS diseases may be due to differences in its mature form, concentration, and local environmental context.

In this review, we discuss IL‐38 signal transduction, its role in physiological niches, and putative mechanisms in pathological contexts. Firstly, we review the molecular biological properties of IL‐38, focusing on its origin, gene and protein expression, as well as biologically active forms and receptors. Secondly, we outline the physiological functions and signaling pathways of IL‐38 and discuss how its activation regulates inflammatory responses and immunity as well as its negative feedback regulatory mechanism. Finally, we turn to a discussion of possible roles and speculative mechanisms of IL‐38 in CNS diseases and possible therapeutic approaches for IL‐38 manipulation in the clinic.

## 2 MOLECULAR BIOLOGY OF IL‐38

### 2.1 Origin, gene, and the protein expression of IL‐38

**FIGURE 1:**
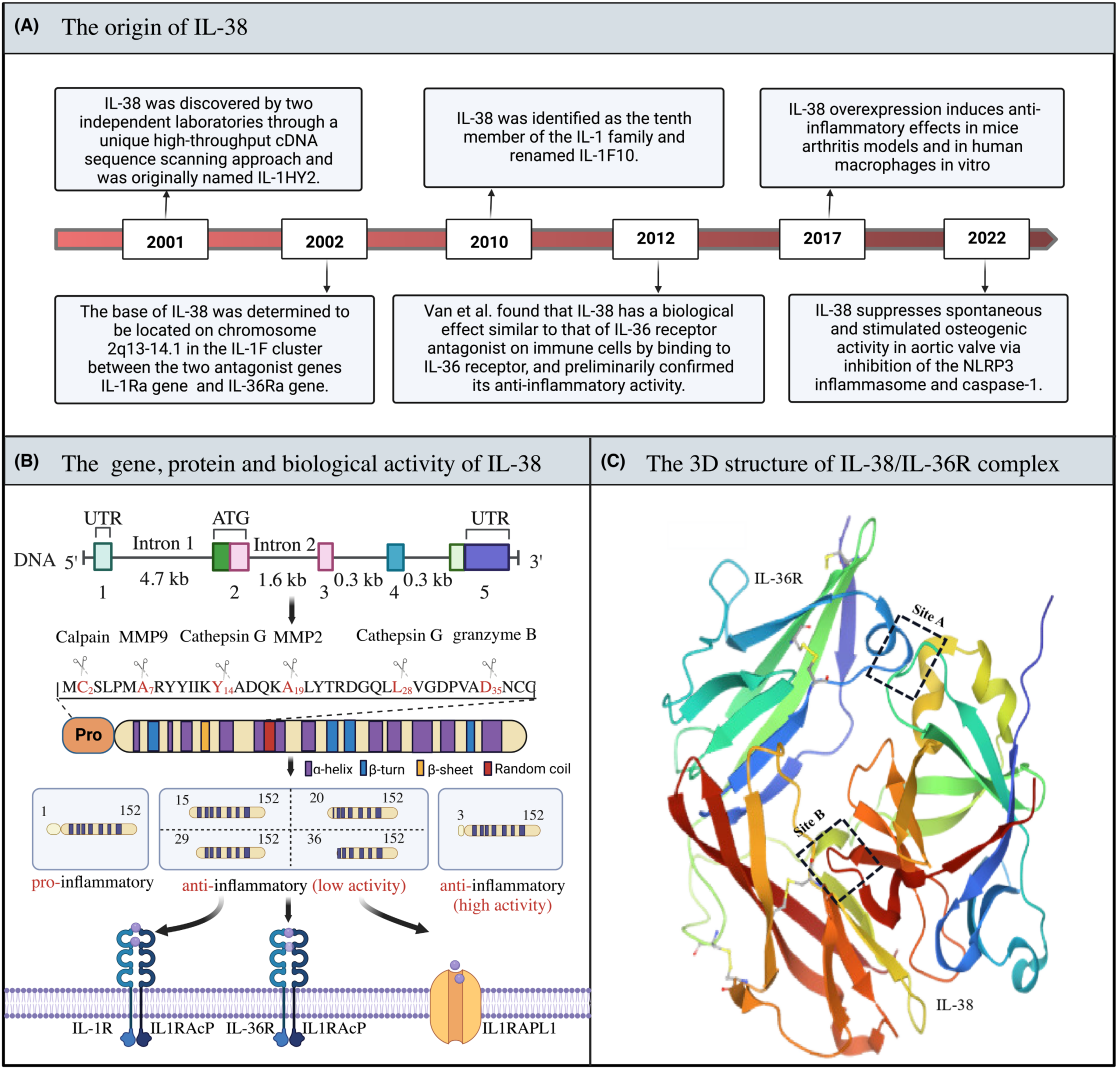
Origin, gene, and the protein structure of IL‐38. (A) The milestone events associated with IL‐38. (B) The IL‐38 gene is located on human chromosome 2q13‐14.1, between the two antagonist genes IL‐1Ra and IL‐36Ra. The IL‐38 gene is mainly composed of 5 exons of 7.8 kb DNA genome, encoding a precursor protein of 152 amino acids (AA) with a molecular weight of 17 kDa. The existence forms of IL‐38 protein can be divided into two categories: IL‐38 full‐length form and IL‐38 mature form. There are many types of mature forms of IL‐38, such as IL‐38 (aa2‐152, aa3‐152, aa5‐152, aa7‐152, and aa20‐152). Due to the lack of a signal peptide and caspase‐1 cleavage site, IL‐38, like most members of IL‐1F, requires N‐terminal cleavage to be biologically active. These predicted cleavage enzymes (e.g., Calpain, MMP2/9, Cathepsin G, Granzyme B) are indicated by arrows with the corresponding cleavage sites. (C) The 3D crystal structure of the IL‐38‐IL‐36R complex is shown. The two critical binding sites have been marked by dotted boxes (black).

IL‐38 is the most newly identified anti‐inflammatory cytokine and belongs to the IL‐36 subgroup of the IL‐1 family (F).^34^ As the IL‐38 gene was successfully cloned by Lin et al. and Gan et al. in 2001 (Figure 1A), it was first confirmed to be a member of the IL‐1 receptor family and named IL‐1HY2 and IL‐1F10.^25^ Previous studies found that, aside from IL‐33 and IL‐18, the genes encoding human IL‐1F members were primarily located on chromosome 2.^35^ Indeed, the IL‐38 gene is located on human chromosome 2q13‐14.1, between the two antagonist genes IL‐1Ra and IL‐36Ra (Figure 1B).^36^ The IL‐38 gene has been found to be mainly composed of 5 exons of a 7.8 kb DNA genome, encoding a 152‐amino acid (AA) precursor protein with a 17 kDa molecular mass. IL‐38 protein is mainly composed of 19 amino acids including alanine, glutamic acid, and leucine, with the highest proportion accounting for approximately 9.2%.^37^ Notably, IL‐38 protein shares 41% homology with IL‐1Ra and 43% homology with IL‐36Ra. Since the encoded proteins have receptor antagonist characteristics, it is speculated that their genes may be derived from the IL‐1F receptor antagonist ancestral gene.^38^ Likewise, the human IL‐38 protein is also composed of 152 amino acids, and its three‐dimensional protein structure has the typical 12‐β chain three‐lobed structure of IL‐1 family cytokines.^39^ Due to the lack of a signal peptide and caspase‐1 cleavage site, IL‐38, like most members of IL‐1F, requires N‐terminal cleavage to be biologically active.^26^

### 2.2 Post‐translational processing, active forms, and biological functions of IL‐38

Based on the fact that most IL‐1 family members acquire activity after N‐terminal processing by proteases, a similar possibility was inferred for IL‐38.^40^, ^41^, ^42^ Six different recombinant forms of human IL‐38 (rhIL‐38) have been identified, including full‐length (aa1‐152), aa2‐152, aa3‐152, aa5‐152, aa7‐152, and aa20‐152.^43^ However, the specific cleavage enzyme for the truncated form of IL‐38 and the corresponding cleavage site are still unclear. IL‐38 has been shown to undergo N‐terminal acetylation modification in apoptotic cells to produce rhIL‐38 20–152 (truncated IL‐38), which has unique biological activities.^44^ Full‐length IL‐38 induces increased IL‐6 production by human macrophages, whereas truncated IL‐38 reduces IL‐6 production by attenuating the c‐Jun N‐terminal kinase (JNK)/activator protein (AP)‐1 pathway downstream of IL1RAPL1. In addition, the forms aa5‐152 and aa20‐152 also match a predicted cleavage site 9 amino acids upstream of an A‐X‐Asp motif (where A is an aliphatic amino acid), which is conserved in the IL‐1 family and is important for the processing of IL‐36 cytokines.^45^ The cleavage site of matrix metallopeptidase (MMP) 2 fits the A‐x‐Asp model of truncated IL‐38, which predicts that MMP2, MMP9, cathepsin G, and granzyme B may be IL‐38 cleavage enzymes.

Irene et al. found that IL‐38 is highly expressed in the brains of patients with ASD stimulated by neuropeptide,^32^ and mainly exists in three different recombinant forms, such as IL‐38 aa 1–152, IL‐38 aa 3–152, and IL‐38 aa 5–152; moreover, IL‐38 aa 3–152 has the strongest inhibitory effect on IL‐1β and chemokine CXCL8, indicating that the mature form of IL‐38 has higher biological activity. Furthermore, some scholars believe that calpain processes the naturally secreted IL‐38 precursor; secreted IL‐38 is cleaved and activated by chymase, granzyme B, and neutrophil elastase during the formation of neutrophil extracellular traps (NETs); moreover, granzyme B is involved in apoptosis. IL‐1α, which regulates gene transcription, is present in the nucleus of apoptotic cells, indicating that IL‐38 may express nuclear localization sequences in apoptotic cells. Although the cleaved form of IL‐38 has been increasingly discovered, a large amount of experimental data is still needed to reveal and elucidate the cleavage site, cleavage enzyme, and biological activity of IL‐38 in the future.

### 2.3 IL‐38 receptors and their putative signaling pathways under a physiological context

There are currently three main types of receptors that have been proven to directly bind to IL‐38, such as IL‐1R1, IL‐36R (also known as IL1R6 or IL‐1Rrp2), and IL‐1RAPL1.^19^, ^33^, ^46^ IL‐1R1 is actually the receptor for IL‐1α and IL‐1β and is not essential for IL‐38 to exert its biological effects.^47^ The well‐established receptor for IL‐38 signaling is IL‐36R. IL‐38 competitively binds to IL‐36R and blocks the IL‐36/IL‐36R signaling pathway, as does IL‐36R antagonist. IL‐38 has pro‐ or anti‐inflammatory bifunctional properties, depending on its concentration, forms expressed, post‐translational processing, and local environmental context.^48^ Low concentrations of IL‐38 protein exert anti‐inflammatory functions by binding to IL‐36R or IL‐1R1 to prevent recruitment of the coreceptor IL‐1 receptor accessory protein (IL‐1RAcP) and/or may recruit inhibitory receptors (e.g., SIGIRR, TIGIRR1, or TIGIRR2), preventing the recruitment of the myeloid differentiation primary response 88 (MyD88) adapter protein, thereby blocking nuclear factor kappa B (NF‐κB) or mitogen‐activated protein kinase (MAPK) signaling.^34^, ^48^, ^49^, ^50^ This feature of IL‐38 is analogous to that of IL‐37, which interacts with IL‐18R to recruit a single immunoglobulin SIGIRR to exert an anti‐inflammatory effect when present at low concentrations but exhibits contrasting functions at high concentrations.^27^ In addition, a third potential receptor for IL‐38 is the inhibitory coreceptor of the IL‐1R family (IL‐1RAPL1). In macrophages in an apoptotic conditioned medium, IL‐38 activates IL‐1RAPL1 to inhibit IL‐6 secretion to block JNK/AP‐1 signaling‐dependent antagonism.^46^ However, the receptor, biological function, and signal transduction pathway of IL‐38 under a pathophysiological context are still unclear, therefore, more experimental data are still needed to solve the aforementioned problems of IL‐38.

## 3 THE PUTATIVE EFFECTS AND MECHANISMS OF IL‐38 UNDERPINNING CNS DISEASES

### 3.1 IL‐38 and neuromyelitis optica disorder

Neuromyelitis optica disorder (NMOD) is a demyelinating autoimmune disease mediated by specific antibodies, with astrocytes representing the main immune target.^51^, ^52^ It is characterized by long‐segment transverse myelitis, severe optic neuritis, and intractable vomiting. It has high morbidity, recurrence, and fatality rates, and is the main cause of severe disability in adults.^51^, ^53^, ^54^ NMOD was once considered a subtype of multiple sclerosis (MS)^55^ and was not separated until the discovery of the specific pathogenic antibody aquaporin 4 (aquaporin 4 Antibody, AQP4‐IgG) in 2004.^56^ AQP4‐IgG in the serum, a highly specific biomarker for NMOD, freely penetrates the blood–brain barrier (BBB) and binds to AQP4 on the foot processes of astrocytes to activate complement as well as natural killer cells and the Fc region of AQP4‐IgG binding. This mediates antibody‐dependent cell cytotoxicity (ADCC), leading to astrocyte degranulation and damage.^57^, ^58^, ^59^ The ability of AQP4 to combine with AQP4‐IgG to cause complement‐dependent cytotoxicity (CDC) and ADCC is achieved through the IgG Fc region binding to complement protein C1q and effector cell receptor FcR, activating the classical complement pathway. This not only causes cellular damage through the formation of the pore‐like membrane attack complex (MAC) but also increases vascular permeability through the production of the complement activators C3a and C5a, upregulating the chemotactic inflammatory response.^60^, ^61^ In addition, the activation of complement and damaged astrocytes can lead to IL‐6 and other cytokine secretion, further damaging the BBB and leading to the infiltration of inflammatory cells such as neutrophils and eosinophils.^62^ Simultaneously, oligodendrocytes are attacked, which eventually leads to myelination, axonal damage, and neuronal necrosis. Therefore, effectively reducing AQP4‐IgG penetration of the BBB and resultant inflammatory response, is a critical measure of NMOD prevention and treatment.

IL‐38 has been shown to limit the inflammatory cascade response in vivo, including the secretion of chemokines involved in the Th17 pathway.^31^ Th17 cells are key drivers of autoimmune diseases, including NMOD.^63^, ^64^ Several studies have shown that AQP4 antigen stimulation polarizes the immune response into a Th17 pool and secretes Th17‐associated cytokines such as IL‐6, IL‐17, and IL‐21.^65^ The production of these proinflammatory cytokines can induce the differentiation of B cells into antibody‐producing plasma cells, thereby promoting the production of serum AQP4 antibodies and interfering with neural function. Additionally, Th17 cells, specific for the expression of AQP4, can destroy the BBB, allowing anti‐AQP4 autoantibodies and activated complement to accumulate at the foci of multinucleated cells through the BBB.^66^, ^67^ Th17 cells may thus serve as target cells for NMOD therapy, highlighting the therapeutic potential of IL‐38.^68^

**FIGURE 2:**
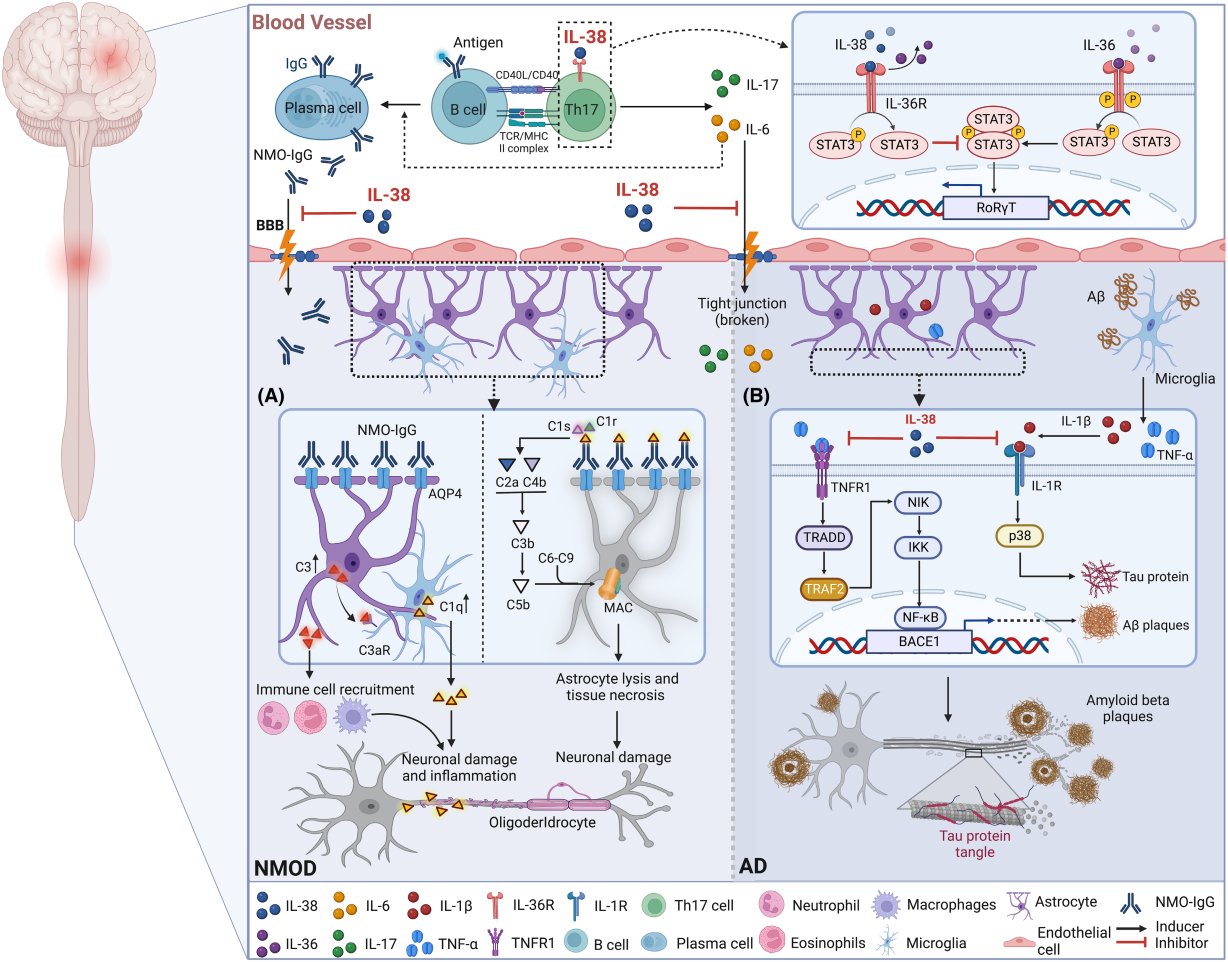
The putative effects and mechanisms of IL‐38 in NMOD and AD. (A) In NMOD, abnormally elevated AQP4‐specific antibody IgG1 in the serum crosses the BBB disrupted by pro‐inflammatory cytokines and enters the brain parenchyma. Subsequently, AQP4‐specific antibodies induce AQP4 internalization by binding to AQP4 on astrocyte terminal foot processes, thereby reducing astrocyte surface AQP4 expression and activating astrocytes. Ultimately, activation of the complement cascade forms the MAC, leading to astrocyte lysis and neuronal necrosis. Notably, IL‐38 can block IL‐36/IL‐36R signaling by competitively antagonizing IL‐36 receptor (IL‐36R) and ILRAPL1 on Th17 cell membranes, thereby inhibiting pro‐inflammatory cytokines (i.e., IL‐17 and IL‐6) expression. Downregulation of IL‐17 and IL‐6 expression levels reduced plasma cell differentiation and antibody production, ultimately alleviating BBB disruption in NMOD. (B) In AD, IL‐38 inhibits Tau phosphorylation and neuronal plaque formation by competing with IL‐1β and TNF‐α for binding to IL‐1R1 and TNFR1, thereby reducing neuronal degeneration and necrosis.

Previous studies have shown that IL‐38 can block the differentiation and function of Th17 cells by inhibiting the NF‐κB and MAPK signaling pathways.^69^ In addition, IL‐38 can significantly increase the expression of Sirtuin 1 (SIRT1) while reducing hypoxia‐inducible factor (HIF)‐1α, AP‐1, and NF‐κB expression, thereby improving the Th17/Treg imbalance in the inflammatory response.^70^ Although the role of IL‐38 in NMOD and its corresponding molecular mechanism has not yet been reported, we speculate that SIRT1/HIF‐1α, NF‐κB, or MAPK signal transduction may play a role in IL‐38 regulation of Th17 cells, thereby protecting against disease progression (Figure 2). Future studies are required to delineate the specific molecular mechanism of IL‐38's protective effect in NMOD to provide a reliable basis for novel therapeutic development in this domain.

### 3.2 IL‐38 and AD

AD is a neurodegenerative disease characterized by progressive memory loss and dementia.^71^, ^72^ The hallmark pathological changes of AD include β‐amyloid (Aβ) deposition, neurofibrillary tangles, and neuronal loss.^73^, ^74^ Neuroinflammation plays an important role in the development and progression of amyloid plaques.^75^, ^76^ Effectively controlling the inflammatory response (i.e., inhibiting the expression of pro‐inflammatory cytokines and increasing that of anti‐inflammatory cytokines) can significantly reduce Aβ deposition; in contrast, a sustained or amplified pro‐inflammatory response (e.g., chemokine expression and inflammatory cell infiltration) can aggravate Aβ deposition.^77^, ^78^ Therefore, targeting neuroinflammation is currently a recognized effective strategy for treating AD patients.

The novel cytokine IL‐38 exerts anti‐inflammatory effects in most diseases, including AD.^79^, ^80^, ^81^ Yahya et al. found that the expression level of IL‐38 in the serum of newly diagnosed AD patients was pronouncedly increased compared with that in the serum of healthy control subjects. Notably, serum IL‐38 expression levels were further increased in elderly male AD patients treated with anti‐inflammatory drugs (memantine), while this phenomenon was not observed in elderly women, indicating that IL‐38 mediates the anti‐inflammatory protective function of memantine in AD and is sex‐dependent.^79^ The results of this study provide a theoretical basis for IL‐38 as a new biological marker for diagnosis, treatment, and prognosis assessment of AD patients. Due to the lack of research on IL‐38 in the treatment of AD, the potential mechanism of IL‐38 in AD remains unclear.

When AD occurs, a large number of microglia and astrocytes in brain tissue are activated, expressing and releasing a large number of pro‐inflammatory cytokines (i.e., IL‐1β, TNF‐α, and IL‐6), which aggravates the neurological deficits.^82^ Previous studies have shown that IL‐38 can largely downregulate LPS‐stimulated IL‐1β and TNF‐α,^83^ two key triggers of AD pathogenesis and progression.^84^, ^85^ On the one hand, in the brain tissue of β‐APP transgenic mice, Aβ activates microglia to overexpress IL‐1β, causing chronic inflammatory damage to the cranial nerves.^86^, ^87^ IL‐1β induces synaptic loss by increasing prostaglandin E2 production, which leads to presynaptic glutamate release and postsynaptic N‐methyl‐d‐aspartate (NMDA) receptor activation.^88^ In the brains of AD patients, binding of IL‐1β to its receptor, which is widely expressed on the surface of astrocytes, induces the proliferation and activation of more astrocytes and expression of a large amount of S100 protein, which in turn, promotes the overgrowth of atrophic axons. Moreover, it can promote astrocytes to express other Aβ‐binding ligands, such as IL‐6, recombinant alpha‐1‐antichymotrypsin (α1‐ACT), apolipoprotein E (ApoE), and complement, which can induce neural plaque formation and exacerbate disease progression.^7^, ^89^, ^90^ Therefore, IL‐38 significantly alleviates IL‐1β‐induced Aβ deposition and reduces lysosomal damage‐activated Aβ fibril‐mediated NOD‐like receptor pyrin domain‐related protein 3 (NLRP3) inflammasome activation by inhibiting microglial polarization following AD.

In parallel, TNF‐α plays an important role in the pathogenesis and development of AD.^91^, ^92^ TNF‐α is expressed at low levels in healthy adult brains, whereas it is overexpressed in neurodegenerative brains.^93^ TNF‐α mediates neuronal apoptosis by binding to the receptors TNFR1 and TNFR2.^94^, ^95^ Numerous studies have shown that in AD brains, the levels of TNFR1 are increased and TNFR2 is decreased,^96^, ^97^, ^98^ indicating that TNFR1 is necessary for Aβ‐induced neuronal apoptosis. TNF‐α binds to TNFR1 on neuronal cell membranes, activates the death domain (DD), and triggers a signaling cascade through the binding of nuclear factor NF‐κB to the BACE1 promoter, stimulating the formation of amyloid plaques.^99^ Therefore, we speculate that inhibiting the inflammatory factor/NF‐κB signaling pathway may represent a promising approach to AD prevention and treatment. Specifically, the use of IL‐38 in AD to reduce the expression of pro‐inflammatory IL‐1β and TNF‐α and inhibit the NF‐κB/BACE1 signaling axis is an interesting direction for future study (Figure 2).^33^

### 3.3 IL‐38 and ASD

ASD is a pervasive neurodevelopmental disorder clinically characterized by deficits in communication and social interactions, as well as the presence of stereotypic behaviors.^100^, ^101^, ^102^ The prevalence of ASD is estimated to be 1 in 54 children in the United States and causes an enormous economic burden.^103^, ^104^ Although significant progress has been made in elucidating the pathogenesis of ASD, the exact etiology remains unclear. In recent years, immune dysfunction and brain inflammation have been considered in the pathogenesis of neuropsychiatric disorders, including ASD.^105^, ^106^, ^107^, ^108^ Several inflammatory molecules, such as IL‐1β, TNF‐α, and CXCL8, have been identified in the brain and cerebrospinal fluid of people with autism. In the pathological process of ASD, stimulation of brain mast cells (MCs) and activation of microglia causes the upregulation of the aforementioned pro‐inflammatory cytokines expression. For instance, adrenocorticotropin‐releasing factor (CRF) is secreted by the hypothalamus under stress and, together with neurotensin (NT), stimulates brain MCs to release inflammatory and neurotoxic mediators that disrupt the BBB.^108^, ^109^ In the meanwhile, mediators from MCs can activate microglia, secreting IL‐1β and CXCL‐8 in response to NT and CRF, causing local inflammation and abnormal connections between neurons in the amygdala, ultimately leading to ASD symptoms.^110^, ^111^ Thus, methods to inhibit inflammation in the amygdala may constitute a new strategy for the treatment of ASD.

**FIGURE 3:**
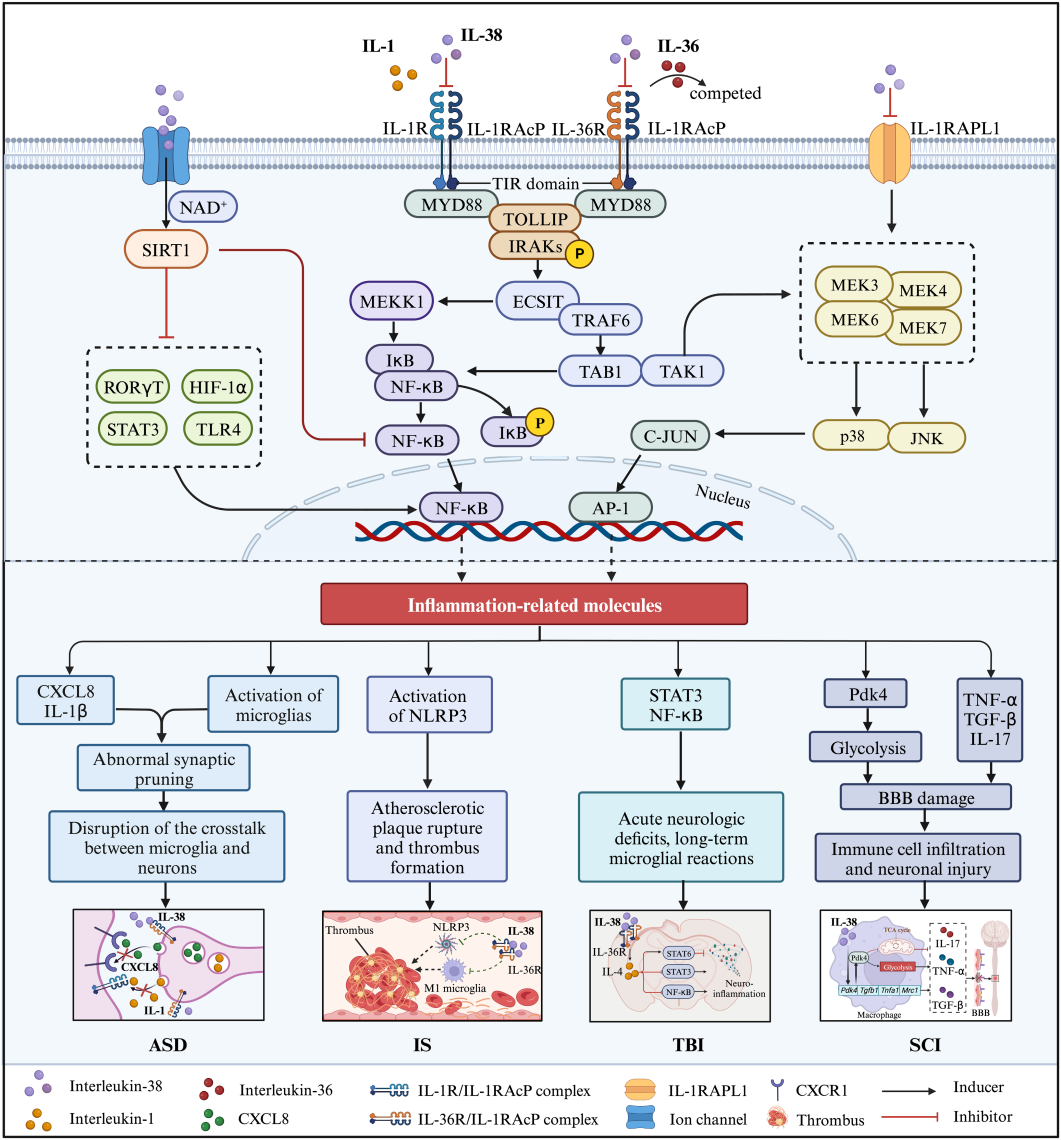
The mechanisms of IL‐38 underpinning in ASD, IS, TBI, and SCI. IL‐38 regulates immune and inflammatory responses after ASD, IS, TBI, and SCI by competitively binding to IL36R with IL‐36. Briefly, IL‐1R1 and IL‐36R are activated by the agonists IL‐1 and IL‐36 (α, γ and β), respectively. The heterodimeric receptor complexes composed of IL‐1R1 or IL‐36R and IL‐1RAcP contribute to myeloid differentiation primary response 88 (MyD88) recruitment through the intracellular toll‐interleukin 1 receptor (TIR) domain, and the downstream signaling pathways that are activated include NF‐κB and MAPK pathways by extracellular regulated protein kinases (ERK), p38 or c‐Jun N‐terminal kinase (JNK), which stimulate the activator protein‐1 (AP‐1). NF‐κB and AP‐1 then bind DNA and stimulate the production of proinflammatory cytokines and chemokines. Conversely, IL‐38 acts as an antagonist to IL‐1R1 and IL‐36R, inhibiting IL‐1RAcP recruitment and agonist binding, similar to what occurs for IL‐1Ra and IL‐36Ra. In addition, IL‐38 selectively recruits MEK or upregulates channel protein‐dependent SIRT1 by binding to IL1RAPL1, thereby reducing NF‐κB, AP‐1, and MAPK, and ultimately regulating the expression of multiple cytokines involved in the pathological process of ASD, IS, TBI, and SCI.

Treatments based on this premise may include anti‐inflammatory molecules, such as IL‐38, which has been reported to inhibit the release of pro‐inflammatory cytokines from MCs and microglia. Recently, Irene Tsilioni et al. found decreased expression of IL‐38 and its receptor IL‐36R in the amygdala of children with ASD, suggesting IL‐38 is associated with ASD pathogenesis. Moreover, IL‐38 inhibited the secretion of pro‐inflammatory molecules in cultured human adult (IL‐1β and CXCL8) and embryonic (CXCL8) microglia upon NT stimulation, especially at concentrations up to 100 ng/mL.^32^ This finding suggests that the IL‐38/IL‐36R axis may be an important pathway in the development of this disease. Specifically, IL‐38 may act as an antagonist to bind to IL‐36R and prevent the activation of downstream microglia, thereby inhibiting the production of inflammatory factors. In previous experiments, IL‐37 treatment was considered to inhibit the secretion and gene expression of IL‐1β and CXCL8 and repair the connections between microglia and neurons.^112^ IL‐38 was shown to possess similar properties to IL‐37 with regard to regulating the activity of microglia and exerted even more of an effect. IL‐38 has an equally strong inhibitory effect on inflammatory mediators produced by MCs (such as IL‐36) and is effective in alleviating the focal inflammatory response in the amygdala,^113^ which contributes to the pathogenesis of ASD. To conclude, the present study indicates the important role of IL‐38 in the inhibition of activation of MCs and microglia, thus reducing disruption of neuronal connectivity and synaptic pruning in the amygdala by neuroinflammation, which supports its development as a potential treatment for ASD (Figure 3).

### 3.4 IL‐38 and IS

Stroke is characterized by high morbidity and mortality, and is the third leading risk factor leading to early death and disability.^114^ Stroke includes ischemic and hemorrhagic stroke types. IS is the most common type of stroke, accounting for 79% of all strokes.^115^, ^116^ The typical pathological basis of IS is atherosclerosis,^117^ and the main secondary pathological changes include bioenergetic failure, Na^+^/K^+^ imbalance across neuronal membranes, mitochondrial failure, oxidative stress, and neuroinflammation.^118^ Neuroinflammation plays an essential role in the development of pathology post‐IS.^119^, ^120^ Excessive or persistent inflammation aggravates secondary pathological damage and functional deficits after IS by inducing a cascade reaction between pro‐inflammatory cytokines. In contrast, effective inhibition of pro‐inflammatory cytokines significantly attenuated IS‐induced neurological deficits. As a novel anti‐inflammatory cytokine, IL‐38 has been shown to play a crucial role in various CNS diseases including but not limited to cerebrovascular diseases. After receiving tissue plasminogen activator (tPA) treatment, the symptoms of pathological damage in IS patients were effectively alleviated, accompanied by a significant increase in serum IL‐38 levels,^121^ which indicates that IL‐38 is negatively correlated with pathological damage after IS, and can be used as an early and reliable predictive marker for stroke prognosis. However, the specific role of IL‐38 after IS and its underlying molecular mechanisms remains unclear.

The main cause of IS is the formation of atherosclerosis. Atherosclerosis is essentially a chronic inflammatory disease, and many immune cells and inflammatory mediators are involved in the development of atherosclerosis.^122^, ^123^ For instance, risk factors such as hyperlipidemia, hypertension, and diabetes can exacerbate inflammatory responses (like suppression of anti‐inflammatory cytokines and enhancement of pro‐inflammatory cytokines) by increasing and activating macrophages, dendritic cells (DCs), and nonspecific memory cells, which in turn promotes the occurrence of atherosclerosis, eventually leading to the occurrence of IS. IL‐38 was shown to prevent atherosclerosis and reduce the incidence and risk of cardiovascular and cerebrovascular events.^20^, ^124^, ^125^, ^126^, ^127^ IL‐38 can suppress inflammation and reduce pathological damage by regulating innate and adaptive immunity.^128^ IL‐38 reduces the infiltration of monocyte‐derived macrophages in the subvascular endothelium, promotes the transformation of pro‐inflammatory M1 macrophages into anti‐inflammatory M2 macrophages, and reduces the release of pro‐inflammatory cytokines IL‐6 and IL‐23.^24^, ^31^ Interestingly, IL‐38 can inhibit the activation of NLRP3 inflammasome in macrophages, thereby suppressing local inflammation.^24^ In addition, DCs play an important role in atherogenesis.^129^ Immature DCs are immune tolerant, while fully mature DCs are immunogenic. Mature DCs can exacerbate endothelial inflammation and atherosclerosis by activating the TNF‐α‐mediated NF‐κB pathway.^130^ IL‐38 was shown to inhibit the maturation of DCs in atherosclerosis by blocking the NF‐κB pathway, thereby increasing the expression of the anti‐inflammatory cytokine IL‐10 and reducing the expression of the pro‐inflammatory cytokines IL‐23, TNF‐α, and IFN‐γ expression.^126^ Further, innate immune system cells such as monocytes, macrophages, and DCs can establish nonspecific immune memory and modulate inflammatory phenotypes through previous exposure to microbial products, a process known as trained immunity. Trained immunity induces an enhanced pro‐inflammatory state in cells, thereby promoting the induction of atherosclerosis.^131^ Activation of the mammalian target of rapamycin (mTOR) and hypoxia‐inducible factor 1α (HIF‐1α) increases glycolysis and promotes epigenetic reprogramming during innate immune memory generation. Numerous studies have shown that IL‐38 can not only down‐regulate HIF‐1α^70^ but also inhibit the expression of β‐glucan‐induced trained immune‐related genes (*Tnfa*, *Nrlp3*, *Hk2*, and *Pfkp*) and inflammation by blocking the mTOR signaling pathway, thereby limiting the inflammatory response in atherosclerosis.^132^

In addition to anti‐inflammatory effects, IL‐38 can increase the stability of atherosclerotic plaques by reducing cell death such as apoptosis and necroptosis. Among them, the apoptosis of smooth muscle cells and macrophages will gradually increase with the progression of atherosclerosis, resulting in the expansion and vulnerability of atherosclerotic plaques, which in turn increases the risk of IS.^133^, ^134^ The B‐cell leukemia/lymphoma‐2 (Bcl‐2) protein family plays an important role in the intrinsic apoptotic pathway. Bcl‐2 is an anti‐apoptotic protein, while Bax is an apoptotic protein.^135^, ^136^ IL‐38 has been shown to inhibit apoptosis by upregulating Bcl‐2 and downregulating Bax, thereby enhancing the survival of vascular endothelial cells, reducing the apoptosis of smooth muscle cells and macrophages in the fibrous cap, and finally attenuating atherosclerosis.^126^, ^137^ This suggests that IL‐38 can inhibit the progression of atherosclerosis by reducing apoptosis through the Bcl‐2/Bax pathway.

In conclusion, IL‐38 may protect against atherosclerosis by regulating immune cells and inflammatory cytokines. Although the role and potential mechanism of IL‐38 after IS have not yet been reported, considering that atherosclerosis is the main cause of IS development, combined with IL‐38 is negatively correlated with pathological damage after IS, we speculate that IL‐38 is likely to affect pathological injury after IS by regulating immune cells, inflammatory response, and apoptosis, however, its specific mechanism remains to be elucidated (Figure 3).

### 3.5 IL‐38 and TBI

TBI is a disease resulting from the destruction and dysfunction of brain tissue caused by external violence.^138^, ^139^ The pathological mechanisms induced by TBI are complex and diverse, including oxidative stress, endoplasmic reticulum stress, cell death, and especially inflammatory response.^140^, ^141^ Appropriate inflammatory responses play a neuro‐defensive effect, while abnormal or excessive inflammatory responses have a damaging effect. For instance, pro‐inflammatory cytokines (i.e., IL‐1β, IL‐17, and IL‐36) are remarkably elevated after TBI, accompanied by severe cognitive impairment.^142^ Conversely, inhibiting the expression of these pro‐inflammatory cytokines can markedly reduce oxidative stress and neuronal death, promote synaptic remodeling, and thereby alleviate neurological dysfunction. Therefore, targeted regulation of inflammatory cytokine expression plays a crucial role in the intervention and treatment of TBI patients. However, how these cytokines, individually and through their interactions, are involved in the pathogenesis of TBI is still not fully understood.

IL‐38, a new member of the IL‐1 family, plays an important role in various diseases by inhibiting the IL‐36/IL‐36R signaling pathway, and so does as an antagonist of IL‐36R.^33^, ^37^, ^143^ It is reported that TBI induces a significant increase in the expression levels of IL‐33, IL‐1β, IL‐38, TNF‐α, IFN‐α, and IL‐19 in the hippocampus 3 h after TBI.^144^ Ethanol intoxication (EI) accompanied by TBI can cause dose‐dependent down‐regulation of IL‐33, IL‐1β, IL‐38, TNF‐α, and IL‐19 (except IFN‐α), as well as selective up‐regulation of IL‐13 and IL‐12, suggesting that these cytokines, including IL‐38, may play an important role in the development of pathology post‐TBI. Mechanistically, neuronal injury experiments in vitro show that ethanol can induce STAT6 phosphorylation and transcriptional activation in acute phase neurons while inhibiting STAT6 expression and activity can prevent the effects of EI on IL‐33 and TNF‐α, but not on IL‐13. In addition, STAT6 inhibition limited the effects of EI on microglial activation and trogliosis and preserved synaptic density and baseline neuronal activity on day 7 post‐TBI. EI plays a significant immunoregulatory role in cytokine induction and microglial activation in patients with TBI, mainly through activation of the STAT6 pathway, ultimately contributing to beneficial results.^145^, ^146^ It has been clarified that the biological function of IL‐38 is equivalent to an antagonist of IL‐36R, and it exerts corresponding biological activities by blocking IL‐36/IL‐36R.^49^, ^69^ IL‐36/IL‐36R signaling induces TH9 differentiation dependent on IL‐4 signaling for efficient induction of STAT6 phosphorylation.^147^

Based on the findings mentioned above, we speculate that IL‐38 is likely to play an important role after TBI, and part of the mechanism may be realized through IL‐36/IL‐36R/IL‐4/STAT6 (Figure 3). However, more experimental data are still needed to verify the role and potential mechanism of IL‐38 after TBI.

### 3.6 IL‐38 and spinal cord injury

SCI is a temporary or permanent injury to the spinal cord, accompanied by motor and neurological dysfunction.^148^, ^149^ SCI can be divided into primary and secondary injuries.^150^ Among them, the causes and mechanisms of secondary injury are diverse, mainly involving microglial activation, reactive oxygen species release, and neuroinflammation.^151^ Neuroinflammation is known to play an essential role in the pathological development of SCI.^152^ In the early stage after SCI, an appropriate inflammatory response can remove cellular debris and limit the spread of damage; in the late stage, an amplified or sustained inflammatory response can aggravate tissue damage and delay the healing of brain tissue.^153^, ^154^ Therefore, strengthening the regulation of the inflammatory environment of the spinal cord will help protect neurons from damage and reduce neurological dysfunction.

Cytokines, as important neuro‐inflammatory mediators, play an essential role in the homeostasis of the inflammatory and immune microenvironment after SCI.^151^, ^155^ For example, SCI induces the expression and release of a large number of pro‐inflammatory cytokines (i.e., IL‐1β, IL‐6, and TNF‐α), which not only aggravates the infiltration of inflammatory cells but also leads to amplified production of cytokines, thereby inducing an inflammatory storm leading to multiple organ failure.^153^, ^156^ In contrast, effectively inhibiting the expression and release of these pro‐inflammatory cytokines not only protects neurons from damage but also accelerates the healing of damaged tissues.

It is understood that IL‐38 is highly expressed in the spinal cord and mainly originates from macrophages and microglia. Arnaud et al. demonstrated that IL‐38 can promote the production of inflammatory mediators (e.g., TNF‐α or TGF‐β1) in a cell‐intrinsic manner in vitro, correlating with the inflammatory signature of SCI in vivo.^17^ Considering that IL‐38 may have similar functions to IL‐33, a member of the same family, it is very likely that IL‐38 also has the characteristic of dual nucleocytoplasmic distribution, although there are still no research reports investigating this. There is evidence that IL‐38 expression is not detected in the supernatants of human macrophages and keratinocytes stimulated by LPS/IFN‐γ,^157^, ^158^, ^159^ indicating that the secretion of IL‐38 may be cell‐ and stressor‐specific. Notably, IL‐38 knockdown reduced the inflammatory response and improved the pathological changes of SCI after SCI by limiting the production of TNF‐α and IL‐17 by macrophages. Additionally, IL‐38 deficiency prevented the upregulation of pyruvate dehydrogenase kinase isozyme 4 (Pdk4) expression in bone marrow‐derived macrophage (BMDM).^17^ Pdk4 is a key metabolic checkpoint in macrophage polarization.^160^, ^161^ Interestingly, the knockout of IL‐38 prevents LPS/IFN‐γ from stimulating the metabolic shift of microglia to the glycolytic pathway by regulating Pdk4 transcription, blocking the transformation of M1 microglia, and enhancing M2 microglial transformation, which in turn reduces peripheral monocyte recruitment, stabilizes local inflammatory storms, and promotes neuronal survival and synaptic remodeling, thereby ameliorating motor deficits after SCI (Figure 3). An in‐depth exploration of the molecular mechanism of IL‐38 regulating Pdk4 transcription and affecting macrophage energy metabolism pathways will provide a theoretical basis for axonal and myelin regeneration, synaptic remodeling, and neurological function repair in SCI.

## 4 IMPLICATIONS FOR IL‐38‐RELATED THERAPEUTIC STRATEGIES

IL‐38, an IL‐36R antagonist, exerts potent anti‐inflammatory effects and represents a promising therapeutic for CNS disease. IL‐38 inhibits the NF‐κB, MAPK (p38, ERK1/2, MEK), STAT1, and STAT3 signaling pathways by competitively binding to IL‐36R, IL‐1R, and IL‐1RAPL1, thereby suppressing the expression of proinflammatory cytokines and chemokines. Notably, in both in vivo and in vitro models of childhood ASD, IL‐38 protein expression levels are markedly decreased in the amygdala and increased in the serum of patients.^32^ Exogenous administration of recombinant IL‐38 has furthermore been shown to exert anti‐inflammatory effects by inhibiting IL‐1β and CXCL8 released by microglia, leading to improved social and communication abilities. In addition to CNS disorders, IL‐38 inhibits cytokine Th17 (IL‐17, IL‐23, IL‐22) and TNF‐ɑ expression in rheumatoid arthritis by reducing macrophage infiltration.^31^, ^128^, ^162^ To date, although no clinical applications of IL‐38 have been reported, functional and mechanistic studies of IL‐38 in a large number of human and animal disease models have highlighted its clinical therapeutic and diagnostic value. For instance, when patients with chronic hepatitis B recovered after treatment, their serum IL‐38 levels were markedly elevated^163^; when patients with IS had been treated with tPA, their serum IL‐38 levels were also dramatically elevated.^121^ It is thus suspected that IL‐38 expression may be a new biological marker for early diagnosis, treatment, and prognostic assessment of patients with hepatitis B, stroke, and various other diseases. Although the anti‐inflammatory function of IL‐38 in systemic tissue and organ disease has been proven, its mechanism of action and clinical application in neurological diseases remain unclear. Given that IL‐38 is a seemingly important therapeutic target for CNS diseases, selective agonists and inhibitors of IL‐38, as well as upstream signaling pathways need to be further explored.

Previous studies have found that the inflammatory factors IL‐17, IL‐22, IL‐36γ, and IFN inhibit terminal differentiation of keratinocytes, accompanied by a decrease in IL‐38 expression. This is likely due to the presence of elements in the IL‐38 promoter that bind to cAMP‐reactive blockers such as ICER.^158^ Thus, selective inhibitors of IL‐17, IL‐22, IL‐36γ, and IFN are likely to indirectly increase the effects of IL‐38 expression. (1) There are three main inhibitors of IL‐17: secukinumab, ixekizumab, and brodalumab.^164^, ^165^, ^166^, ^167^ The former two are anti‐IL‐17A monoclonal antibodies, which can directly reduce the expression and activity of free IL‐17A by specifically binding to IL‐17A to form an antigen–antibody immune complex. The latter is a monoclonal antibody against the IL‐17A receptor that inhibits the physiological function of IL‐17 by targeting IL‐17R and blocking its signaling. The three listed IL‐17 inhibitors are mainly used in the treatment of psoriasis, and they are in phase I, II, and III clinical trials. A previous study found that after 8 weeks of secukinumab treatment in patients with psoriasis, expression levels of IL‐38 at both the mRNA and protein levels were significantly increased in skin lesion tissue. This indicates that IL‐17 inhibitors can regulate and potentially agonize IL‐38.^158^ Although these three IL‐17A inhibitors are expected to be potential IL‐38 agonists, the mechanism by which this occurs remains to be demonstrated. (2) There are two inhibitors of IL‐36, spesolimab, and imsidolimab, which target the IL‐36 receptor and block the binding of IL‐36γ mainly through competitive inhibition of the receptor.^168^, ^169^ Predictive analysis of potential transcription factor‐binding sites on the IL‐38 and IL‐36Ra promoter sequences revealed the presence of elements in the IL‐38 promoter that may bind to cAMP‐responsive blockers, including ICER and Kruppel‐like Factor 4 (KLF4). These are known to be induced by inflammatory cytokines and are associated with keratinocyte differentiation.^170^, ^171^ ICER is a cAMP‐responsive element binding protein (CREB), the most potent transcriptional repressor that blocks CRE‐dependent gene transcription. KLF4 is also a restriction transcription factor induced by inflammatory factors, including IL‐36γ. IL‐36γ triggers IL‐38 downregulation in dedifferentiated keratin‐forming cells by agonizing repressive transcription factors at the IL‐38 promoter. Consequently, inhibition of IL‐36γ relieves the antagonistic effect on IL‐38, thereby indirectly increasing the IL‐38 expression and activity. Interestingly, the inhibitory effect of IL‐36γ on IL‐38 in keratinocytes was mild compared to IL‐17 and IL‐22. Considering IL‐38 competitively binds to IL‐36 receptors to inhibit the IL‐36/IL‐36R signaling pathway, this suggests the presence of a feedback regulation mechanism between the cytokines.^158^ Therefore, how these two IL‐36 inhibitors specifically antagonize IL‐38 remains to be explored in depth. (3) The inhibitors of IL‐22 and IFN, fezakinumab and emapalumab, respectfully, downregulate the expression of the corresponding cytokine by binding to and neutralizing them.^172^, ^173^ Given that IL‐38 levels are also strongly downregulated by IL‐22 and, to a lesser extent, by IFN‐γ in keratinocytes, it is likely that inhibitors of both have an indirect role in agonizing IL‐38.

Collectively, excessive or persistent inflammation is a robust contributor to the deterioration and pathological changes associated with CNS diseases. Effectively increasing the expression of anti‐inflammatory cytokines or inhibiting the expression of pro‐inflammatory cytokines is an ideal strategy for the prevention and treatment of CNS diseases. As an important anti‐inflammatory cytokine, IL‐38 plays an essential role in CNS diseases (i.e., NMOD, AD, ASD, IS, TBI, and SCI). For instance, serum IL‐38 expression levels are markedly decreased in patients with cerebral IS, accompanied by increased neurological deficits. After treatment with tPA, serum IL‐38 levels increased dramatically, while neurological functioning improved. Given the negative correlation between IL‐38 and stroke‐induced neurological deficits, IL‐38 may serve as a biomarker for early diagnosis of this disease.^121^, ^174^ This is just one example of many that have been discussed in this review, highlighting the neuroprotective role IL‐38 may play in CNS diseases. Despite the increasing clinical applications of IL‐38, the role and potential mechanisms of IL‐38 in CNS diseases are incompletely characterized due to an insufficient understanding of its biological characteristics. Although some regulators of IL‐38 and its upstream signaling pathways have been discovered, specific inhibitors or agonists are still lacking. Increased research in this domain will assist in better understanding the pathophysiological function and molecular mechanism of IL‐38, which will further provide support for IL‐38 as a novel target for the treatment of CNS diseases (Table 1).

**TABLE 1:** The potential agonists of IL‐38.

Classification	Compound	Disease type	Targets for molecules or pathways	Efficiency	References
IL‐17 inhibitors	Secukinumab	Ankylosing spondylitis, psoriasis and psoriatic arthritis	The expression of IL‐38 is indirectly inhibited by specifically binding to IL‐17A to form an antigen–antibody immune complex	++	157, 163, 164
	Ixekizumab				157, 163, 165
	Brodalumab		By blocking the IL‐17 receptor, the inflammatory cytokine IL‐17A is down‐regulated, and the expression of IL‐38 is indirectly inhibited		157, 163, 166
IL‐36γ inhibitors	Spesolimab	Palmoplantar pustulosis, generalized pustular psoriasis, Ulcerative colitis, Crohn's disease and hidradenitis supurativa	Block subsequent activation of the IL‐36 receptor, thereby blocking the IL‐38 signaling pathway	+	157, 167, 169, 170
	Imsidolimab	Acne vulgaris, hidradenitis supurativa and generalized pustular psoriasis			157, 168, 169, 170
IL‐22 inhibitor	Fezakinumab	Atopic dermatitis	The expression of IL‐38 is indirectly inhibited through negative feedback by neutralizing IL‐22	++	157, 171
IFN‐γ Inhibitor	Emapalumab	Hemophagocytic lymphohistiocytosis	A fully human immunoglobulin G1 monoclonal antibody that noncompetitively inhibits IFN‐γ, thereby inhibiting the expression of IL‐38	+	157, 172

## 5 CONCLUSIONS AND FUTURE PERSPECTIVES

IL‐38 is now generally accepted as playing a key anti‐inflammatory protective role in a variety of diseases, including central nervous system diseases. As summarized in this review, a large amount of compelling evidence supports the notion that targeted regulation of IL‐38 expression can provide promising new options for the treatment of many neuroinflammation‐related diseases. As our understanding of the IL‐38‐regulating pathway (i.e., IL‐36/IL‐36R, NF‐κB/Th17/IL‐17, and IL‐1β/CXCL8) continues to uncover new drug targets, IL‐38 modulators may provide new opportunities for developing treatments for many currently incurable diseases. Thus far, most information has been obtained utilizing mouse models. To what degree these observations are applicable to humans remains unknown. Clinical trials using antibody‐targeting IL‐38 have recently been initiated in pneumonia and autoimmune diseases, and treatment with recombinant IL‐38 could be useful in other diseases (e.g., IS and ASD). In the future, fully elucidating the concentration, post‐translationally modified cleavage sites, cleavage enzymes, mature forms, biological activities, and pathophysiological functions of IL‐38 would further facilitate the development of therapies that modulate IL‐38/inflammation signaling to treat CNS diseases (Table 2).

**TABLE 2:** The role of IL‐38 in CNS diseases.

Central nervous diseases	Receptors that IL‐38 binds to	The possible effects of IL‐38	The underlying mechanism of IL‐38	References
Neuromyelitis optica disorder (NMOD)	IL‐1RAPL1 IL‐23R	On the one hand, IL‐38 inhibits the production of AQP4 antibodies induced by Th17 cells; on the other hand, IL‐38 may decrease pro‐inflammatory factors such as IL‐6, IL‐17, and IL‐23 secreted by Th17 cells	IL‐38/MAPKs/NF‐κB/Th17 axis IL‐38/SIRT1/HIF‐1α/Th17 axis	50, 51, 52, 53, 54, 55, 56, 57, 58, 59, 60, 61, 62, 63, 64, 65, 66, 67, 68, 69
Alzheimer's disease (AD)	IL‐36R IL‐1R TNFR1	IL‐38 decreased neuronal fiber plaque (Aβ) production and Tau protein phosphorylation by down‐regulating the expression of IL‐1β and TNF‐α released from microglia	IL‐38/36R/NF‐κB/BACE1 axis IL‐38/1R/NF‐κB/BACE1 axis IL‐38/TNFR1/NF‐κB/BACE1 axis	70, 71, 72, 73, 74, 75, 76, 77, 78, 79, 80, 81, 82, 83, 84, 85, 86, 87, 88, 89, 90, 91, 92, 93, 94, 95, 96, 97, 98
Autism spectrum disorder (ASD)	IL‐36R	IL‐38 may inhibit IL‐1β and CXCL8 secretion and gene expression in microglia and repair abnormal neuronal pruning	IL‐38/36R/CXCL8/IL‐1β axis	99, 100, 101, 102, 103, 104, 105, 106, 107, 108, 109, 110, 111, 112
Ischemic stroke (IS)	IL‐1R1	IL‐38 clears ischemic blood vessels in the stroke‐damaged vessels while accelerating vascular endothelial proliferation and repair of blood vessels	IL‐38/IL‐1R1/SIRT1/HIF‐1α/axis	113, 114, 115, 116, 117, 118, 119, 120, 121, 122, 123, 124, 125, 126, 127, 128, 129, 130, 131, 132, 133, 134, 135, 136
Traumatic brain injury (TBI)	IL‐36R	IL‐38 may inhibit the expression of STAT3 and NF‐κB while activating STAT6, alleviating the acute neurologic defects induced by TBI and the long‐term microglia reaction	IL‐38/IL‐36R/STAT6 axis	137, 138, 139, 140, 141, 142, 143, 144, 145, 146
Spinal cord injury (SCI)	–	In macrophages, IL‐38 can up‐regulate the transcription of Pdk4, promoting macrophage glycolysis and the secretion of TNF‐α, TGF‐β, and IL‐17. These inflammatory factors in turn destroy the BBB, causing immune cells to enter the central nervous system and damage spinal cord neurons	IL‐38/Pdk4/TNF‐α axis	147, 148, 149, 150, 151, 152, 153, 154, 155, 156, 157, 158, 159, 160

## AUTHOR CONTRIBUTIONS

Yuan Gao conceived and designed the work. Luwei Cai, Yuan Gao, and Yulu Wu were responsible for writing the whole passage. Yidan Zhang, Wenjing Reng, Lili Li, Yirui Song, Ziguang Lei, Youzhuang Wu, Luwen Zhu, Min Jiang, Jing Li, and Dongya Li were in charge of checking and revision of the manuscript. All the figures in the article were made by Luwei Cai. Chengliang Luo, Guohong Li, and Luyang Tao are the guarantors of this work.

## FUNDING INFORMATION

This work was supported by the National Natural Science Foundation of China (82101972, 81530062, 81971800, 81971163, 82271409, and 82001382), NIH grant (NS119538, Li, G), the Jiangsu Provincial Natural Science Foundation of China (SBK2020040785), the Suzhou Municipal Science and Technology Bureau (SYS2019027), the China Postdoctoral Science Foundation (2020M681723), the Jiangsu Province Postdoctoral Science Support Program (2021K590C), the Undergraduate Training Program for Innovation and Entrepreneurship, Soochow University (202110285044Z), a project of invigorating healthcare through science, technology, and education (KJXW2019018), and a project funded by the Priority Academic Program Development of Jiangsu Higher Education Institutions (PAPD).

## CONFLICT OF INTEREST STATEMENT

The authors declare no competing interests.

## Data Availability

Data sharing not applicable to this article as no datasets were generated or analysed during the current study.

## References

[1] Piancone F , La Rosa F , Marventano I , Saresella M , Clerici M . The role of the inflammasome in neurodegenerative diseases. Molecules. 2021;26(4):953. 33670164 10.3390/molecules26040953 PMC7916884

[2] Xu J , Ma C , Hua M , Li J , Xiang Z , Wu J . CNS and CNS diseases in relation to their immune system. Front Immunol. 2022;13:1063928. 36466889 10.3389/fimmu.2022.1063928 PMC9708890

[3] Malani Shukla N , Lotze TE , Muscal E . Inflammatory diseases of the central nervous system. Neurol Clin. 2021;39(3):811‐828. 34215388 10.1016/j.ncl.2021.04.004

[4] Schain M , Kreisl WC . Neuroinflammation in neurodegenerative disorders‐a review. Curr Neurol Neurosci Rep. 2017;17(3):25. 28283959 10.1007/s11910-017-0733-2

[5] Liu Z , Cheng X , Zhong S , et al. Peripheral and central nervous system immune response crosstalk in amyotrophic lateral sclerosis. Front Neurosci. 2020;14:575. 32612503 10.3389/fnins.2020.00575 PMC7308438

[6] Kölliker‐Frers R , Udovin L , Otero‐Losada M , et al. Neuroinflammation: an integrating overview of reactive‐Neuroimmune cell interactions in health and disease. Mediators Inflamm. 2021;2021:9999146. 34158806 10.1155/2021/9999146 PMC8187052

[7] Lopez‐Rodriguez AB , Hennessy E , Murray CL , et al. Acute systemic inflammation exacerbates neuroinflammation in Alzheimer's disease: IL‐1β drives amplified responses in primed astrocytes and neuronal network dysfunction. Alzheimers Dement. 2021;17(10):1735‐1755. 34080771 10.1002/alz.12341 PMC8874214

[8] Tanzi RE . Alzheimer's disease risk and the interleukin‐1 genes. Ann Neurol. 2000;47(3):283‐285. 10716246

[9] Lonnemann N , Hosseini S , Ohm M , et al. IL‐37 expression reduces acute and chronic neuroinflammation and rescues cognitive impairment in an Alzheimer's disease mouse model. Elife. 2022;11:e75889. 36040311 10.7554/eLife.75889 PMC9481244

[10] Clausen BH , Wirenfeldt M , Høgedal SS , et al. Characterization of the TNF and IL‐1 systems in human brain and blood after ischemic stroke. Acta Neuropathol Commun. 2020;8(1):81. 32503645 10.1186/s40478-020-00957-y PMC7273684

[11] Zhang SR , Nold MF , Tang SC , et al. IL‐37 increases in patients after ischemic stroke and protects from inflammatory brain injury, motor impairment and lung infection in mice. Sci Rep. 2019;9(1):6922. 31061403 10.1038/s41598-019-43364-7 PMC6502884

[12] Lindblad C , Rostami E , Helmy A . Interleukin‐1 receptor antagonist as therapy for traumatic brain injury. Neurotherapeutics. 2023;20(6):1508‐1528. 37610701 10.1007/s13311-023-01421-0 PMC10684479

[13] Becher B , Spath S , Goverman J . Cytokine networks in neuroinflammation. Nat Rev Immunol. 2017;17(1):49‐59. 27916979 10.1038/nri.2016.123

[14] Lauritano D , Mastrangelo F , D'Ovidio C , et al. Activation of mast cells by neuropeptides: the role of pro‐inflammatory and anti‐inflammatory cytokines. Int J Mol Sci. 2023;24(5):4811. 36902240 10.3390/ijms24054811 PMC10002992

[15] Baker JR , Fenwick PS , Koss CK , et al. IL‐36 receptor agonist and antagonist imbalance drives neutrophilic inflammation in COPD. JCI Insight. 2022;7(15):e155581. 35763349 10.1172/jci.insight.155581 PMC9462491

[16] Diaz‐Barreiro A , Huard A , Palmer G . Multifaceted roles of IL‐38 in inflammation and cancer. Cytokine. 2022;151:155808. 35066449 10.1016/j.cyto.2022.155808

[17] Huard A , Do HN , Frank AC , et al. IL‐38 ablation reduces local inflammation and disease severity in experimental autoimmune encephalomyelitis. J Immunol. 2021;206(5):1058‐1066. 33504620 10.4049/jimmunol.2000923

[18] Shi L , Ye H , Huang J , et al. IL‐38 exerts anti‐inflammatory and antifibrotic effects in thyroid‐associated ophthalmopathy. J Clin Endocrinol Metab. 2021;106(8):e3125‐e3142. 33693700 10.1210/clinem/dgab154

[19] Li Z , Ding Y , Peng Y , et al. Effects of IL‐38 on macrophages and myocardial ischemic injury. Front Immunol. 2022;13:894002. 35634320 10.3389/fimmu.2022.894002 PMC9136064

[20] Lai M , Peng H , Wu X , Chen X , Wang B , Su X . IL‐38 in modulating hyperlipidemia and its related cardiovascular diseases. Int Immunopharmacol. 2022;108:108876. 35623295 10.1016/j.intimp.2022.108876

[21] Li H , Zhu L , Wang R , et al. Therapeutic effect of IL‐38 on experimental autoimmune uveitis: reprogrammed immune cell landscape and reduced Th17 cell pathogenicity. Invest Ophthalmol Vis Sci. 2021;62(15):31. 10.1167/iovs.62.15.31 PMC8727319 34967854

[22] Xu WD , Su LC , Fu L , et al. IL‐38, a potential therapeutic agent for lupus, inhibits lupus progression. Inflamm Res. 2022;71(7–8):963‐975. 35776155 10.1007/s00011-022-01581-3

[23] Wei Q , Chen X , Chen X , Yuan Z , Wang C . Contribution of IL‐38 in lung immunity during *Pseudomonas aeruginosa*‐induced pneumonia. Shock. 2022;57(5):703‐713. 35583912 10.1097/SHK.0000000000001919

[24] Ge Y , Chen J , Hu Y , Chen X , Huang M . IL‐38 alleviates inflammation in sepsis in mice by inhibiting macrophage apoptosis and activation of the NLRP3 inflammasome. Mediators Inflamm. 2021;2021:6370911. 34955683 10.1155/2021/6370911 PMC8709774

[25] Lin H , Ho AS , Haley‐Vicente D , et al. Cloning and characterization of IL‐1HY2, a novel interleukin‐1 family member. J Biol Chem. 2001;276(23):20597‐20602. 11278614 10.1074/jbc.M010095200

[26] van de Veerdonk FL , de Graaf DM , Joosten LA , Dinarello CA . Biology of IL‐38 and its role in disease. Immunol Rev. 2018;281(1):191‐196. 29247986 10.1111/imr.12612

[27] Xie L , Huang Z , Li H , Liu X , Zheng S , Su W . IL‐38: a new player in inflammatory autoimmune disorders. Biomolecules. 2019;9(8):345. 31387327 10.3390/biom9080345 PMC6723600

[28] de Graaf DM , Jaeger M , van den Munckhof ICL , et al. Reduced concentrations of the B cell cytokine interleukin 38 are associated with cardiovascular disease risk in overweight subjects. Eur J Immunol. 2021;51(3):662‐671. 33125159 10.1002/eji.201948390 PMC7983920

[29] Conti P , Pregliasco FE , Bellomo RG , et al. Mast cell cytokines IL‐1, IL‐33, and IL‐36 mediate skin inflammation in psoriasis: a novel therapeutic approach with the anti‐inflammatory cytokines IL‐37, IL‐38, and IL‐1Ra. Int J Mol Sci. 2021;22(15):8076. 34360845 10.3390/ijms22158076 PMC8348737

[30] Shaik Y , Sabatino G , Maccauro G , et al. IL‐36 receptor antagonist with special emphasis on IL‐38. Int J Immunopathol Pharmacol. 2013;26(1):27‐36. 23527706 10.1177/039463201302600103

[31] Boutet MA , Najm A , Bart G , et al. IL‐38 overexpression induces anti‐inflammatory effects in mice arthritis models and in human macrophages in vitro. Ann Rheum Dis. 2017;76(7):1304‐1312. 28288964 10.1136/annrheumdis-2016-210630

[32] Tsilioni I , Pantazopoulos H , Conti P , Leeman SE , Theoharides TC . IL‐38 inhibits microglial inflammatory mediators and is decreased in amygdala of children with autism spectrum disorder. Proc Natl Acad Sci USA. 2020;117(28):16475‐16480. 32601180 10.1073/pnas.2004666117 PMC7368277

[33] van de Veerdonk FL , Stoeckman AK , Wu G , et al. IL‐38 binds to the IL‐36 receptor and has biological effects on immune cells similar to IL‐36 receptor antagonist. Proc Natl Acad Sci USA. 2012;109(8):3001‐3005. 22315422 10.1073/pnas.1121534109 PMC3286950

[34] Garlanda C , Dinarello CA , Mantovani A . The interleukin‐1 family: back to the future. Immunity. 2013;39(6):1003‐1018. 24332029 10.1016/j.immuni.2013.11.010 PMC3933951

[35] Gibson MS , Fife M , Bird S , Salmon N , Kaiser P . Identification, cloning, and functional characterization of the IL‐1 receptor antagonist in the chicken reveal important differences between the chicken and mammals. J Immunol. 2012;189(2):539‐550. 22689884 10.4049/jimmunol.1103204

[36] Bensen JT , Dawson PA , Mychaleckyj JC , Bowden DW . Identification of a novel human cytokine gene in the interleukin gene cluster on chromosome 2q12‐14. J Interferon Cytokine Res. 2001;21(11):899‐904. 11747621 10.1089/107999001753289505

[37] Yuan X , Peng X , Li Y , Li M . Role of IL‐38 and its related cytokines in inflammation. Mediators Inflamm. 2015;2015:807976. 25873772 10.1155/2015/807976 PMC4383490

[38] Nicklin MJ , Barton JL , Nguyen M , FitzGerald MG , Duff GW , Kornman K . A sequence‐based map of the nine genes of the human interleukin‐1 cluster. Genomics. 2002;79(5):718‐725. 11991722 10.1006/geno.2002.6751

[39] Fields JK , Günther S , Sundberg EJ . Structural basis of IL‐1 family cytokine signaling. Front Immunol. 2019;10:1412. 31281320 10.3389/fimmu.2019.01412 PMC6596353

[40] Aoyagi T , Newstead MW , Zeng X , Kunkel SL , Kaku M , Standiford TJ . IL‐36 receptor deletion attenuates lung injury and decreases mortality in murine influenza pneumonia. Mucosal Immunol. 2017;10(4):1043‐1055. 27966554 10.1038/mi.2016.107 PMC5471142

[41] Towne JE , Renshaw BR , Douangpanya J , et al. Interleukin‐36 (IL‐36) ligands require processing for full agonist (IL‐36α, IL‐36β, and IL‐36γ) or antagonist (IL‐36Ra) activity. J Biol Chem. 2011;286(49):42594‐42602. 21965679 10.1074/jbc.M111.267922 PMC3234937

[42] Henry CM , Sullivan GP , Clancy DM , Afonina IS , Kulms D , Martin SJ . Neutrophil‐derived proteases escalate inflammation through activation of IL‐36 family cytokines. Cell Rep. 2016;14(4):708‐722. 26776523 10.1016/j.celrep.2015.12.072

[43] de Graaf DM , Maas RJA , Smeekens SP , et al. Human recombinant interleukin‐38 suppresses inflammation in mouse models of local and systemic disease. Cytokine. 2021;137:155334. 33128926 10.1016/j.cyto.2020.155334 PMC7725974

[44] Mora J , Schlemmer A , Wittig I , et al. Interleukin‐38 is released from apoptotic cells to limit inflammatory macrophage responses. J Mol Cell Biol. 2016;8(5):426‐438. 26892022 10.1093/jmcb/mjw006

[45] Dinarello CA . Overview of the IL‐1 family in innate inflammation and acquired immunity. Immunol Rev. 2018;281(1):8‐27. 29247995 10.1111/imr.12621 PMC5756628

[46] Pavlowsky A , Zanchi A , Pallotto M , et al. Neuronal JNK pathway activation by IL‐1 is mediated through IL1RAPL1, a protein required for development of cognitive functions. Commun Integr Biol. 2010;3(3):245‐247. 20714405 10.4161/cib.3.3.11414 PMC2918768

[47] Steiger S . Targeting IL‐1 receptor signaling in AKI. J Am Soc Nephrol. 2023;34(10):1601‐1603. 37782544 10.1681/ASN.0000000000000215 PMC10564364

[48] de Graaf DM , Teufel LU , Joosten LAB , Dinarello CA . Interleukin‐38 in health and disease. Cytokine. 2022;152:155824. 35220115 10.1016/j.cyto.2022.155824

[49] Andoh A , Nishida A . Pro‐ and anti‐inflammatory roles of interleukin (IL)‐33, IL‐36, and IL‐38 in inflammatory bowel disease. J Gastroenterol. 2023;58(2):69‐78. 36376594 10.1007/s00535-022-01936-x

[50] Dowling JP , Nikitin PA , Shen F , et al. IL‐38 blockade induces anti‐tumor immunity by abrogating tumor‐mediated suppression of early immune activation. MAbs. 2023;15(1):2212673. 37216961 10.1080/19420862.2023.2212673 PMC10208126

[51] Jarius S , Paul F , Weinshenker BG , Levy M , Kim HJ , Wildemann B . Neuromyelitis optica. Nat Rev Dis Primers. 2020;6(1):85. 33093467 10.1038/s41572-020-0214-9

[52] Uzawa A , Mori M , Kuwabara S . MOG antibody disorders and AQP4 antibody NMO spectrum disorders share a common immunopathogenesis. J Neurol Neurosurg Psychiatry. 2018;89(9):900. 29875185 10.1136/jnnp-2018-318231

[53] Wingerchuk DM , Lennon VA , Lucchinetti CF , Pittock SJ , Weinshenker BG . The spectrum of neuromyelitis optica. Lancet Neurol. 2007;6(9):805‐815. 17706564 10.1016/S1474-4422(07)70216-8

[54] Levy M , Fujihara K , Palace J . New therapies for neuromyelitis optica spectrum disorder. Lancet Neurol. 2021;20(1):60‐67. 33186537 10.1016/S1474-4422(20)30392-6

[55] Filippi M , Bar‐Or A , Piehl F , et al. Multiple sclerosis. Nat Rev Dis Primers. 2018;4(1):43. 30410033 10.1038/s41572-018-0041-4

[56] Lennon VA , Wingerchuk DM , Kryzer TJ , et al. A serum autoantibody marker of neuromyelitis optica: distinction from multiple sclerosis. Lancet. 2004;364(9451):2106‐2112. 15589308 10.1016/S0140-6736(04)17551-X

[57] Weinshenker BG , Wingerchuk DM . Neuromyelitis optica: clinical syndrome and the NMO‐IgG autoantibody marker. Curr Top Microbiol Immunol. 2008;318:343‐356. 18219825 10.1007/978-3-540-73677-6_14

[58] Howe CL , Kaptzan T , Magaña SM , Ayers‐Ringler JR , LaFrance‐Corey RG , Lucchinetti CF . Neuromyelitis optica IgG stimulates an immunological response in rat astrocyte cultures. Glia. 2014;62(5):692‐708. 24492996 10.1002/glia.22635 PMC5392242

[59] Chen T , Lennon VA , Liu YU , et al. Astrocyte‐microglia interaction drives evolving neuromyelitis optica lesion. J Clin Invest. 2020;130(8):4025‐4038. 32568214 10.1172/JCI134816 PMC7410082

[60] Carnero Contentti E , Correale J . Neuromyelitis optica spectrum disorders: from pathophysiology to therapeutic strategies. J Neuroinflammation. 2021;18(1):208. 34530847 10.1186/s12974-021-02249-1 PMC8444436

[61] Zipfel PF , Skerka C . Complement regulators and inhibitory proteins. Nat Rev Immunol. 2009;9(10):729‐740. 19730437 10.1038/nri2620

[62] Araki M . Blockade of IL‐6 signaling in neuromyelitis optica. Neurochem Int. 2019;130:104315. 30342072 10.1016/j.neuint.2018.10.012

[63] Uzawa A , Mori M , Kuwabara S . Cytokines and chemokines in neuromyelitis optica: pathogenetic and therapeutic implications. Brain Pathol. 2014;24(1):67‐73. 24345220 10.1111/bpa.12097 PMC8029308

[64] Maciak K , Pietrasik S , Dziedzic A , et al. Th17‐related cytokines as potential discriminatory markers between neuromyelitis optica (Devic's disease) and multiple sclerosis‐a review. Int J Mol Sci. 2021;22(16):8946. 34445668 10.3390/ijms22168946 PMC8396435

[65] Chen H , Tang X , Li J , et al. IL‐17 crosses the blood‐brain barrier to trigger neuroinflammation: a novel mechanism in nitroglycerin‐induced chronic migraine. J Headache Pain. 2022;23(1):1. 34979902 10.1186/s10194-021-01374-9 PMC8903553

[66] Bukhari W , Barnett MH , Prain K , Broadley SA . Molecular pathogenesis of neuromyelitis optica. Int J Mol Sci. 2012;13(10):12970‐12993. 23202933 10.3390/ijms131012970 PMC3497307

[67] Linhares UC , Schiavoni PB , Barros PO , et al. The ex vivo production of IL‐6 and IL‐21 by CD4+ T cells is directly associated with neurological disability in neuromyelitis optica patients. J Clin Immunol. 2013;33(1):179‐189. 22948743 10.1007/s10875-012-9780-2

[68] Chihara N , Yamamura T . Immuno‐pathogenesis of neuromyelitis optica and emerging therapies. Semin Immunopathol. 2022;44(5):599‐610. 35635574 10.1007/s00281-022-00941-9

[69] Han Y , Mora J , Huard A , et al. IL‐38 ameliorates skin inflammation and limits IL‐17 production from γδ T cells. Cell Rep. 2019;27(3):835‐846.e835. 30995480 10.1016/j.celrep.2019.03.082

[70] Pei B , Chen K , Zhou S , Min D , Xiao W . IL‐38 restrains inflammatory response of collagen‐induced arthritis in rats via SIRT1/HIF‐1α signaling pathway. Biosci Rep. 2020;40(5):BSR20182431. 32347300 10.1042/BSR20182431 PMC7256678

[71] Scheltens P , De Strooper B , Kivipelto M , et al. Alzheimer's disease. Lancet. 2021;397(10284):1577‐1590. 33667416 10.1016/S0140-6736(20)32205-4 PMC8354300

[72] Breijyeh Z , Karaman R . Comprehensive review on Alzheimer's disease: causes and treatment. Molecules. 2020;25(24):5789. 33302541 10.3390/molecules25245789 PMC7764106

[73] Ozben T , Ozben S . Neuro‐inflammation and anti‐inflammatory treatment options for Alzheimer's disease. Clin Biochem. 2019;72:87‐89. 30954437 10.1016/j.clinbiochem.2019.04.001

[74] Zhang H , Wei W , Zhao M , et al. Interaction between Aβ and tau in the pathogenesis of Alzheimer's disease. Int J Biol Sci. 2021;17(9):2181‐2192. 34239348 10.7150/ijbs.57078 PMC8241728

[75] Jorfi M , Maaser‐Hecker A , Tanzi RE . The neuroimmune axis of Alzheimer's disease. Genome Med. 2023;15(1):6. 36703235 10.1186/s13073-023-01155-w PMC9878767

[76] Parhizkar S , Holtzman DM . APOE mediated neuroinflammation and neurodegeneration in Alzheimer's disease. Semin Immunol. 2022;59:101594. 35232622 10.1016/j.smim.2022.101594 PMC9411266

[77] Kummer MP , Ising C , Kummer C , et al. Microglial PD‐1 stimulation by astrocytic PD‐L1 suppresses neuroinflammation and Alzheimer's disease pathology. EMBO J. 2021;40(24):e108662. 34825707 10.15252/embj.2021108662 PMC8672180

[78] Mehla J , Singh I , Diwan D , et al. STAT3 inhibitor mitigates cerebral amyloid angiopathy and parenchymal amyloid plaques while improving cognitive functions and brain networks. Acta Neuropathol Commun. 2021;9(1):193. 34911575 10.1186/s40478-021-01293-5 PMC8672532

[79] Yahya AM , Zuhair R . Interleukin‐38 (IL‐38) is a novel biochemical marker in sera of Iraqi patients with Alzheimers disease. Biochem. Cell. Arch. 2021;21(1):1579‐1584.

[80] The E , de Graaf DM , Zhai Y , et al. Interleukin 38 alleviates aortic valve calcification by inhibition of NLRP3. Proc Natl Acad Sci USA. 2022;119(36):e2202577119. 36037361 10.1073/pnas.2202577119 PMC9457240

[81] Wang Q , Ma L , An C , Wise SG , Bao S . The role of IL‐38 in intestinal diseases – its potential as a therapeutic target. Front Immunol. 2022;13:1051787. 36405715 10.3389/fimmu.2022.1051787 PMC9670310

[82] Leng F , Edison P . Neuroinflammation and microglial activation in Alzheimer disease: where do we go from here? Nat Rev Neurol. 2021;17(3):157‐172. 33318676 10.1038/s41582-020-00435-y

[83] Yuan XL , Li Y , Pan XH , Zhou M , Gao QY , Li MC . Production of recombinant human interleukin‐38 and its inhibitory effect on the expression of proinflammatory cytokines in THP‐1 cells. Mol Biol (Mosk). 2016;50(3):466‐473. 27414784 10.7868/S0026898416030137

[84] Cheng X , Shen Y , Li R . Targeting TNF: a therapeutic strategy for Alzheimer's disease. Drug Discov Today. 2014;19(11):1822‐1827. 24998784 10.1016/j.drudis.2014.06.029

[85] Singhal G , Jaehne EJ , Corrigan F , Toben C , Baune BT . Inflammasomes in neuroinflammation and changes in brain function: a focused review. Front Neurosci. 2014;8:315. 25339862 10.3389/fnins.2014.00315 PMC4188030

[86] Cotman CW , Tenner AJ , Cummings BJ . Beta‐amyloid converts an acute phase injury response to chronic injury responses. Neurobiol Aging. 1996;17(5):723‐731. 8892345 10.1016/0197-4580(96)00117-0

[87] Tiwari S , Atluri V , Kaushik A , Yndart A , Nair M . Alzheimer's disease: pathogenesis, diagnostics, and therapeutics. Int J Nanomedicine. 2019;14:5541‐5554. 31410002 10.2147/IJN.S200490 PMC6650620

[88] Mishra A , Kim HJ , Shin AH , Thayer SA . Synapse loss induced by interleukin‐1β requires pre‐ and post‐synaptic mechanisms. J Neuroimmune Pharmacol. 2012;7(3):571‐578. 22311599 10.1007/s11481-012-9342-7 PMC3415563

[89] Sheng JG , Mrak RE , Rovnaghi CR , Kozlowska E , Van Eldik LJ , Griffin WS . Human brain S100 beta and S100 beta mRNA expression increases with age: pathogenic implications for Alzheimer's disease. Neurobiol Aging. 1996;17(3):359‐363. 8725896 10.1016/0197-4580(96)00037-1

[90] Shaftel SS , Griffin WS , O'Banion MK . The role of interleukin‐1 in neuroinflammation and Alzheimer disease: an evolving perspective. J Neuroinflammation. 2008;5:7. 18302763 10.1186/1742-2094-5-7 PMC2335091

[91] Jayaraman A , Htike TT , James R , Picon C , Reynolds R . TNF‐mediated neuroinflammation is linked to neuronal necroptosis in Alzheimer's disease hippocampus. Acta Neuropathol Commun. 2021;9(1):159. 34625123 10.1186/s40478-021-01264-w PMC8501605

[92] Torres‐Acosta N , O'Keefe JH , O'Keefe EL , Isaacson R , Small G . Therapeutic potential of TNF‐α inhibition for Alzheimer's disease prevention. J Alzheimers Dis. 2020;78(2):619‐626. 33016914 10.3233/JAD-200711 PMC7739965

[93] Cheng X , Yang L , He P , Li R , Shen Y . Differential activation of tumor necrosis factor receptors distinguishes between brains from Alzheimer's disease and non‐demented patients. J Alzheimers Dis. 2010;19(2):621‐630. 20110607 10.3233/JAD-2010-1253 PMC3746510

[94] Alam MS , Otsuka S , Wong N , et al. TNF plays a crucial role in inflammation by signaling via T cell TNFR2. Proc Natl Acad Sci USA. 2021;118(50):e2109972118. 34873037 10.1073/pnas.2109972118 PMC8685675

[95] Prada JP , Wangorsch G , Kucka K , Lang I , Dandekar T , Wajant H . A systems‐biology model of the tumor necrosis factor (TNF) interactions with TNF receptor 1 and 2. Bioinformatics. 2021;37(5):669‐676. 32991680 10.1093/bioinformatics/btaa844

[96] Li R , Yang L , Lindholm K , et al. Tumor necrosis factor death receptor signaling cascade is required for amyloid‐beta protein‐induced neuron death. J Neurosci. 2004;24(7):1760‐1771. 14973251 10.1523/JNEUROSCI.4580-03.2004 PMC6730458

[97] Perry RT , Collins JS , Wiener H , Acton R , Go RC . The role of TNF and its receptors in Alzheimer's disease. Neurobiol Aging. 2001;22(6):873‐883. 11754994 10.1016/s0197-4580(01)00291-3

[98] Steeland S , Gorlé N , Vandendriessche C , et al. Counteracting the effects of TNF receptor‐1 has therapeutic potential in Alzheimer's disease. EMBO Mol Med. 2018;10(4):e8300. 29472246 10.15252/emmm.201708300 PMC5887909

[99] Wyss‐Coray T , Mucke L . Inflammation in neurodegenerative disease–a double‐edged sword. Neuron. 2002;35(3):419‐432. 12165466 10.1016/s0896-6273(02)00794-8

[100] Lord C , Elsabbagh M , Baird G , Veenstra‐Vanderweele J . Autism spectrum disorder. Lancet. 2018;392(10146):508‐520. 30078460 10.1016/S0140-6736(18)31129-2 PMC7398158

[101] Gyawali S , Patra BN . Autism spectrum disorder: trends in research exploring etiopathogenesis. Psychiatry Clin Neurosci. 2019;73(8):466‐475. 31077508 10.1111/pcn.12860

[102] Jiang CC , Lin LS , Long S , et al. Signalling pathways in autism spectrum disorder: mechanisms and therapeutic implications. Signal Transduct Target Ther. 2022;7(1):229. 35817793 10.1038/s41392-022-01081-0 PMC9273593

[103] Xu G , Strathearn L , Liu B , Bao W . Prevalence of autism spectrum disorder among US children and adolescents, 2014‐2016. JAMA. 2018;319(1):81‐82. 29297068 10.1001/jama.2017.17812 PMC5833544

[104] Maenner MJ , Shaw KA , Baio J , et al. Prevalence of autism spectrum disorder among children aged 8 years – autism and developmental disabilities monitoring network, 11 sites, United States, 2016. MMWR Surveill Summ. 2020;69(4):1‐12. 10.15585/mmwr.ss6904a1 PMC7119644 32214087

[105] Roe K . Autism Spectrum disorder initiation by inflammation‐facilitated neurotoxin transport. Neurochem Res. 2022;47(5):1150‐1165. 35050480 10.1007/s11064-022-03527-x

[106] Matta SM , Hill‐Yardin EL , Crack PJ . The influence of neuroinflammation in autism spectrum disorder. Brain Behav Immun. 2019;79:75‐90. 31029798 10.1016/j.bbi.2019.04.037

[107] Meltzer A , Van de Water J . The role of the immune system in autism Spectrum disorder. Neuropsychopharmacology. 2017;42(1):284‐298. 27534269 10.1038/npp.2016.158 PMC5143489

[108] Theoharides TC , Tsilioni I , Patel AB , Doyle R . Atopic diseases and inflammation of the brain in the pathogenesis of autism spectrum disorders. Transl Psychiatry. 2016;6(6):e844. 27351598 10.1038/tp.2016.77 PMC4931610

[109] Theoharides TC , Stewart JM , Panagiotidou S , Melamed I . Mast cells, brain inflammation and autism. Eur J Pharmacol. 2016;778:96‐102. 25941080 10.1016/j.ejphar.2015.03.086

[110] Sharma SR , Gonda X , Tarazi FI . Autism spectrum disorder: classification, diagnosis and therapy. Pharmacol Ther. 2018;190:91‐104. 29763648 10.1016/j.pharmthera.2018.05.007

[111] Gzielo K , Nikiforuk A . Astroglia in autism spectrum disorder. Int J Mol Sci. 2021;22(21):11544. 34768975 10.3390/ijms222111544 PMC8583956

[112] Tsilioni I , Patel AB , Pantazopoulos H , et al. IL‐37 is increased in brains of children with autism spectrum disorder and inhibits human microglia stimulated by neurotensin. Proc Natl Acad Sci USA. 2019;116(43):21659‐21665. 31591201 10.1073/pnas.1906817116 PMC6815178

[113] Theoharides TC , Kavalioti M , Tsilioni I . Mast cells, stress, fear and autism spectrum disorder. Int J Mol Sci. 2019;20(15):3611. 31344805 10.3390/ijms20153611 PMC6696098

[114] Benjamin EJ , Virani SS , Callaway CW , et al. Heart disease and stroke statistics‐2018 update: a report from the American Heart Association. Circulation. 2018;137(12):e67‐e492. 29386200 10.1161/CIR.0000000000000558

[115] Feske SK . Ischemic stroke. Am J Med. 2021;134(12):1457‐1464. 34454905 10.1016/j.amjmed.2021.07.027

[116] Rabinstein AA . Update on treatment of acute ischemic stroke. Continuum (Minneap Minn). 2020;26(2):268‐286. 32224752 10.1212/CON.0000000000000840

[117] Donnan GA , Fisher M , Macleod M , Davis SM . Stroke. Lancet. 2008;371(9624):1612‐1623. 18468545 10.1016/S0140-6736(08)60694-7

[118] Mendelson SJ , Prabhakaran S . Diagnosis and management of transient ischemic attack and acute ischemic stroke: a review. JAMA. 2021;325(11):1088‐1098. 33724327 10.1001/jama.2020.26867

[119] Gelderblom M , Leypoldt F , Steinbach K , et al. Temporal and spatial dynamics of cerebral immune cell accumulation in stroke. Stroke. 2009;40(5):1849‐1857. 19265055 10.1161/STROKEAHA.108.534503

[120] Chu HX , Kim HA , Lee S , et al. Immune cell infiltration in malignant middle cerebral artery infarction: comparison with transient cerebral ischemia. J Cereb Blood Flow Metab. 2014;34(3):450‐459. 24326388 10.1038/jcbfm.2013.217 PMC3948121

[121] Zare Rafie M , Esmaeilzadeh A , Ghoreishi A , Tahmasebi S , Faghihzadeh E , Elahi R . IL‐38 as an early predictor of the ischemic stroke prognosis. Cytokine. 2021;146:155626. 34157522 10.1016/j.cyto.2021.155626

[122] Frostegård J . Immunity, atherosclerosis and cardiovascular disease. BMC Med. 2013;11:117. 23635324 10.1186/1741-7015-11-117 PMC3658954

[123] Gisterå A , Hansson GK . The immunology of atherosclerosis. Nat Rev Nephrol. 2017;13(6):368‐380. 28392564 10.1038/nrneph.2017.51

[124] Zhang XH , Li Y , Zhou L , Tian GP . Interleukin‐38 in atherosclerosis. Clin Chim Acta. 2022;536:86‐93. 36150521 10.1016/j.cca.2022.09.017

[125] Esmaeilzadeh A , Pouyan S , Erfanmanesh M . Is interleukin‐38 a key player cytokine in atherosclerosis immune gene therapy? Med Hypotheses. 2019;125:139‐143. 30902143 10.1016/j.mehy.2019.02.048

[126] Wei Y , Lan Y , Zhong Y , et al. Interleukin‐38 alleviates cardiac remodelling after myocardial infarction. J Cell Mol Med. 2020;24(1):371‐384. 31746138 10.1111/jcmm.14741 PMC6933378

[127] Zhong Y , Yu K , Wang X , Wang X , Ji Q , Zeng Q . Elevated plasma IL‐38 concentrations in patients with acute ST‐segment elevation myocardial infarction and their dynamics after reperfusion treatment. Mediators Inflamm. 2015;2015:490120. 26819499 10.1155/2015/490120 PMC4706979

[128] Xu WD , Su LC , He CS , Huang AF . Plasma interleukin‐38 in patients with rheumatoid arthritis. Int Immunopharmacol. 2018;65:1‐7. 30268016 10.1016/j.intimp.2018.09.028

[129] Zernecke A . Dendritic cells in atherosclerosis: evidence in mice and humans. Arterioscler Thromb Vasc Biol. 2015;35(4):763‐770. 25675999 10.1161/ATVBAHA.114.303566

[130] Gao W , Liu H , Yuan J , et al. Exosomes derived from mature dendritic cells increase endothelial inflammation and atherosclerosis via membrane TNF‐α mediated NF‐κB pathway. J Cell Mol Med. 2016;20(12):2318‐2327. 27515767 10.1111/jcmm.12923 PMC5134386

[131] Netea MG , Joosten LA , Latz E , et al. Trained immunity: a program of innate immune memory in health and disease. Science. 2016;352(6284):aaf1098. 27102489 10.1126/science.aaf1098 PMC5087274

[132] de Graaf DM , Teufel LU , van de Veerdonk FL , et al. IL‐38 prevents induction of trained immunity by inhibition of mTOR signaling. J Leukoc Biol. 2021;110(5):907‐915. 33620105 10.1002/JLB.3A0220-143RRR PMC8380748

[133] Bennett MR , Sinha S , Owens GK . Vascular smooth muscle cells in atherosclerosis. Circ Res. 2016;118(4):692‐702. 26892967 10.1161/CIRCRESAHA.115.306361 PMC4762053

[134] Duan H , Zhang Q , Liu J , et al. Suppression of apoptosis in vascular endothelial cell, the promising way for natural medicines to treat atherosclerosis. Pharmacol Res. 2021;168:105599. 33838291 10.1016/j.phrs.2021.105599

[135] Peña‐Blanco A , García‐Sáez AJ . Bax, Bak and beyond – mitochondrial performance in apoptosis. FEBS J. 2018;285(3):416‐431. 28755482 10.1111/febs.14186

[136] Banjara S , Suraweera CD , Hinds MG , Kvansakul M . The Bcl‐2 family: ancient origins, conserved structures, and divergent mechanisms. Biomolecules. 2020;10(1):128. 31940915 10.3390/biom10010128 PMC7022251

[137] Jiang L , Zhou X , Huang C , et al. The elevated expression of IL‐38 serves as an anti‐inflammatory factor in osteoarthritis and its protective effect in osteoarthritic chondrocytes. Int Immunopharmacol. 2021;94:107489. 33774357 10.1016/j.intimp.2021.107489

[138] Capizzi A , Woo J , Verduzco‐Gutierrez M . Traumatic brain injury: an overview of epidemiology, pathophysiology, and medical management. Med Clin North Am. 2020;104(2):213‐238. 32035565 10.1016/j.mcna.2019.11.001

[139] Bailes JE , Borlongan CV . Traumatic brain injury. CNS Neurosci Ther. 2020;26(6):593‐594. 32452140 10.1111/cns.13397 PMC7248541

[140] Brett BL , Gardner RC , Godbout J , Dams‐O'Connor K , Keene CD . Traumatic brain injury and risk of neurodegenerative disorder. Biol Psychiatry. 2022;91(5):498‐507. 34364650 10.1016/j.biopsych.2021.05.025 PMC8636548

[141] Simon DW , McGeachy MJ , Bayır H , Clark RS , Loane DJ , Kochanek PM . The far‐reaching scope of neuroinflammation after traumatic brain injury. Nat Rev Neurol. 2017;13(3):171‐191. 28186177 10.1038/nrneurol.2017.13 PMC5675525

[142] Jia Y , Wang G , Ye Y , et al. Niche cells crosstalk in neuroinflammation after traumatic brain injury. Int J Biol Sci. 2021;17(1):368‐378. 33390856 10.7150/ijbs.52169 PMC7757042

[143] Zhang M , Zhou JX , Huang CQ , et al. IL‐38 alleviates airway remodeling in chronic asthma via blocking the profibrotic effect of IL‐36γ. Clin Exp Immunol. 2023;uxad099. 10.1093/cei/uxad099 PMC10719219 37586814

[144] Olde Heuvel F , Holl S , Chandrasekar A , et al. STAT6 mediates the effect of ethanol on neuroinflammatory response in TBI. Brain Behav Immun. 2019;81:228‐246. 31207335 10.1016/j.bbi.2019.06.019

[145] Xu B , Chandrasekar A , Olde Heuvel F , et al. Ethanol intoxication alleviates the inflammatory response of remote organs to experimental traumatic brain injury. Int J Mol Sci. 2020;21(21):8181. 33142949 10.3390/ijms21218181 PMC7663496

[146] Chandrasekar A , Aksan B , Heuvel FO , et al. Neuroprotective effect of acute ethanol intoxication in TBI is associated to the hierarchical modulation of early transcriptional responses. Exp Neurol. 2018;302:34‐45. 29306704 10.1016/j.expneurol.2017.12.017

[147] Harusato A , Abo H , Ngo VL , et al. IL‐36γ signaling controls the induced regulatory T cell‐Th9 cell balance via NFκB activation and STAT transcription factors. Mucosal Immunol. 2017;10(6):1455‐1467. 28327619 10.1038/mi.2017.21 PMC5610052

[148] Hewson DW , Bedforth NM , Hardman JG . Spinal cord injury arising in anaesthesia practice. Anaesthesia. 2018;73(Suppl 1):43‐50. 29313911 10.1111/anae.14139

[149] Anjum A , Yazid MD , Fauzi Daud M , et al. Spinal cord injury: pathophysiology, multimolecular interactions, and underlying recovery mechanisms. Int J Mol Sci. 2020;21(20):7533. 33066029 10.3390/ijms21207533 PMC7589539

[150] Sterner RC , Sterner RM . Immune response following traumatic spinal cord injury: pathophysiology and therapies. Front Immunol. 2022;13:1084101. 36685598 10.3389/fimmu.2022.1084101 PMC9853461

[151] Hellenbrand DJ , Quinn CM , Piper ZJ , Morehouse CN , Fixel JA , Hanna AS . Inflammation after spinal cord injury: a review of the critical timeline of signaling cues and cellular infiltration. J Neuroinflammation. 2021;18(1):284. 34876174 10.1186/s12974-021-02337-2 PMC8653609

[152] Freyermuth‐Trujillo X , Segura‐Uribe JJ , Salgado‐Ceballos H , Orozco‐Barrios CE , Coyoy‐Salgado A . Inflammation: a target for treatment in spinal cord injury. Cells. 2022;11(17):2692. 36078099 10.3390/cells11172692 PMC9454769

[153] Li Y , Ritzel RM , Khan N , et al. Delayed microglial depletion after spinal cord injury reduces chronic inflammation and neurodegeneration in the brain and improves neurological recovery in male mice. Theranostics. 2020;10(25):11376‐11403. 33052221 10.7150/thno.49199 PMC7545988

[154] Li Y , Lei Z , Ritzel RM , et al. Impairment of autophagy after spinal cord injury potentiates neuroinflammation and motor function deficit in mice. Theranostics. 2022;12(12):5364‐5388. 35910787 10.7150/thno.72713 PMC9330534

[155] Xu GY , Xu S , Zhang YX , et al. Cell‐free extracts from human fat tissue with a hyaluronan‐based hydrogel attenuate inflammation in a spinal cord injury model through M2 microglia/microphage polarization. Small. 2022;18(17):e2107838. 35333441 10.1002/smll.202107838

[156] Parvin S , Williams CR , Jarrett SA , Garraway SM . Spinal cord injury increases pro‐inflammatory cytokine expression in kidney at acute and sub‐chronic stages. Inflammation. 2021;44(6):2346‐2361. 34417952 10.1007/s10753-021-01507-x PMC8616867

[157] Zhou H , Zhao Q , Yue C , et al. Interleukin‐38 promotes skin tumorigenesis in an IL‐1Rrp2‐dependent manner. EMBO Rep. 2022;23(6):e53791. 35578812 10.15252/embr.202153791 PMC9171418

[158] Mercurio L , Morelli M , Scarponi C , et al. IL‐38 has an anti‐inflammatory action in psoriasis and its expression correlates with disease severity and therapeutic response to anti‐IL‐17A treatment. Cell Death Dis. 2018;9(11):1104. 30377293 10.1038/s41419-018-1143-3 PMC6207563

[159] Talabot‐Ayer D , Mermoud L , Borowczyk J , et al. Interleukin‐38 interacts with destrin/actin‐depolymerizing factor in human keratinocytes. PLoS One. 2019;14(11):e0225782. 31770407 10.1371/journal.pone.0225782 PMC6879167

[160] Han X , Ma W , Zhu Y , Sun X , Liu N . Advanced glycation end products enhance macrophage polarization to the M1 phenotype via the HIF‐1α/PDK4 pathway. Mol Cell Endocrinol. 2020;514:110878. 32464167 10.1016/j.mce.2020.110878

[161] Min BK , Park S , Kang HJ , et al. Pyruvate dehydrogenase kinase is a metabolic checkpoint for polarization of macrophages to the M1 phenotype. Front Immunol. 2019;10:944. 31134063 10.3389/fimmu.2019.00944 PMC6514528

[162] Takenaka SI , Kaieda S , Kawayama T , et al. IL‐38: a new factor in rheumatoid arthritis. Biochem Biophys Rep. 2015;4:386‐391. 29124228 10.1016/j.bbrep.2015.10.015 PMC5669445

[163] Wang HJ , Jiang YF , Wang XR , Zhang ML , Gao PJ . Elevated serum interleukin‐38 level at baseline predicts virological response in telbivudine‐treated patients with chronic hepatitis B. World J Gastroenterol. 2016;22(18):4529‐4537. 27182162 10.3748/wjg.v22.i18.4529 PMC4858634

[164] Amin M , Darji K , No DJ , Bhutani T , Wu JJ . Review of IL‐17 inhibitors for psoriasis. J Dermatolog Treat. 2018;29(4):347‐352. 29058501 10.1080/09546634.2017.1395796

[165] Baeten D , Sieper J , Braun J , et al. Secukinumab, an interleukin‐17A inhibitor, in ankylosing spondylitis. N Engl J Med. 2015;373(26):2534‐2548. 26699169 10.1056/NEJMoa1505066

[166] Craig S , Warren RB . Ixekizumab for the treatment of psoriasis: up‐to‐date. Expert Opin Biol Ther. 2020;20(6):549‐557. 32050819 10.1080/14712598.2020.1729736

[167] Lebwohl M , Strober B , Menter A , et al. Phase 3 studies comparing brodalumab with ustekinumab in psoriasis. N Engl J Med. 2015;373(14):1318‐1328. 26422722 10.1056/NEJMoa1503824

[168] Blair HA . Spesolimab: first approval. Drugs. 2022;82(17):1681‐1686. 36418672 10.1007/s40265-022-01801-4 PMC9744699

[169] Iznardo H , Puig L . Exploring the role of IL‐36 cytokines as a new target in psoriatic disease. Int J Mol Sci. 2021;22(9):4344. 33919434 10.3390/ijms22094344 PMC8122427

[170] Feinberg MW , Cao Z , Wara AK , Lebedeva MA , Senbanerjee S , Jain MK . Kruppel‐like factor 4 is a mediator of proinflammatory signaling in macrophages. J Biol Chem. 2005;280(46):38247‐38258. 16169848 10.1074/jbc.M509378200

[171] Mead JR , Hughes TR , Irvine SA , Singh NN , Ramji DP . Interferon‐gamma stimulates the expression of the inducible cAMP early repressor in macrophages through the activation of casein kinase 2. A potentially novel pathway for interferon‐gamma‐mediated inhibition of gene transcription. J Biol Chem. 2003;278(20):17741‐17751. 12609974 10.1074/jbc.M301602200

[172] Guttman‐Yassky E , Brunner PM , Neumann AU , et al. Efficacy and safety of fezakinumab (an IL‐22 monoclonal antibody) in adults with moderate‐to‐severe atopic dermatitis inadequately controlled by conventional treatments: a randomized, double‐blind, phase 2a trial. J Am Acad Dermatol. 2018;78(5):872‐881.e876. 29353025 10.1016/j.jaad.2018.01.016 PMC8711034

[173] Vallurupalli M , Berliner N . Emapalumab for the treatment of relapsed/refractory hemophagocytic lymphohistiocytosis. Blood. 2019;134(21):1783‐1786. 31537529 10.1182/blood.2019002289 PMC8938935

[174] Zhu H , Hu S , Li Y , et al. Interleukins and ischemic stroke. Front Immunol. 2022;13:828447. 35173738 10.3389/fimmu.2022.828447 PMC8841354

